# β-aminoisobutyrics acid, a metabolite of BCAA, activates the AMPK/Nrf-2 pathway to prevent ferroptosis and ameliorates lung ischemia-reperfusion injury

**DOI:** 10.1186/s10020-023-00729-z

**Published:** 2023-12-04

**Authors:** Ziyue Zhang, Xingbing Li, Jingwen Guo, Bo He, Lianpan Wu, Rongpei Yang, Xingyue Li, Dandong Fang, XiaoLi Yang, Donghai Yang, Fengxian Wang, Ming Tang, Yu Han, Pedro A. Jose, Hongyong Wang, Chunyu Zeng

**Affiliations:** 1 Department of Cardiology, Daping Hospital, The Third Military Medical University (Army Medical University), Chongqing, P. R. China; 2 Key Laboratory of Geriatric Cardiovascular and Cerebrovascular Disease Research, Chongqing Key Laboratory for Hypertension Research, Chongqing Cardiovascular Clinical Research Center, Ministry of Education of China, Chongqing Institute of Cardiology, Chongqing, P. R. China; 3 Outpatient Department, Hospital of PLA, Hanzhong, Shanxi 96608 P. R. China; 4 Department of Cardiology, Chongqing Hospital of Traditional Chinese Medicine, Chongqing, P. R. China; 5 Division of Renal Diseases & Hypertension, Department of Medicine, Department of Physiology/Pharmacology, The George Washington University School of Medicine & Health Sciences, Washington, DC USA; 6 State Key Laboratory of Trauma, Burns and Combined Injury, Daping Hospital, The Third Military Medical University (Army Medical University), Chongqing, P. R. China; 7 Cardiovascular Research Center of Chongqing College, Chinese Academy of Sciences, University of Chinese Academy of Sciences, Chongqing, P. R. China

**Keywords:** L-BAIBA, Lung ischemia-reperfusion injury, Ferroptosis, Branched chain amino acid, Nrf-2, AMPK

## Abstract

**Background:**

Lung ischemia-reperfusion (I/R) injury is a serious clinical problem without effective treatment. Enhancing branched-chain amino acids (BCAA) metabolism can protect against cardiac I/R injury, which may be related to bioactive molecules generated by BCAA metabolites. L-β-aminoisobutyric acid (L-BAIBA), a metabolite of BCAA, has multi-organ protective effects, but whether it protects against lung I/R injury is unclear.

**Methods:**

To assess the protective effect of L-BAIBA against lung I/R injury, an animal model was generated by clamping the hilum of the left lung, followed by releasing the clamp in C57BL/6 mice. Mice with lung I/R injury were pre-treated or post-treated with L-BAIBA (150 mg/kg/day), given by gavage or intraperitoneal injection. Lung injury was assessed by measuring lung edema and analyzing blood gases. Inflammation was assessed by measuring proinflammatory cytokines in bronchoalveolar lavage fluid (BALF), and neutrophil infiltration of the lung was measured by myeloperoxidase activity. Molecular biological methods, including western blot and immunofluorescence, were used to detect potential signaling mechanisms in A549 and BEAS-2B cells.

**Results:**

We found that L-BAIBA can protect the lung from I/R injury by inhibiting ferroptosis, which depends on the up-regulation of the expressions of GPX4 and SLC7A11 in C57BL/6 mice. Additionally, we demonstrated that the Nrf-2 signaling pathway is key to the inhibitory effect of L-BAIBA on ferroptosis in A549 and BEAS-2B cells. L-BAIBA can induce the nuclear translocation of Nrf-2. Interfering with the expression of Nrf-2 eliminated the protective effect of L-BAIBA on ferroptosis. A screening of potential signaling pathways revealed that L-BAIBA can increase the phosphorylation of AMPK, and compound C can block the Nrf-2 nuclear translocation induced by L-BAIBA. The presence of compound C also blocked the protective effects of L-BAIBA on lung I/R injury in C57BL/6 mice.

**Conclusions:**

Our study showed that L-BAIBA protects against lung I/R injury via the AMPK/Nrf-2 signaling pathway, which could be a therapeutic target.

**Supplementary Information:**

The online version contains supplementary material available at 10.1186/s10020-023-00729-z.

## Graphical Abstract

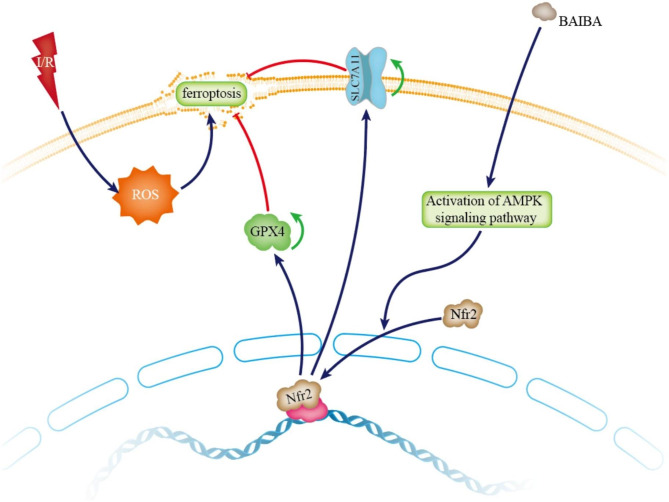

L-BAIBA upregulates the expression of GPX4 and SLC7A11 by activating the AMPK/Nrf-2/GPX4/SLC7A11 signaling pathway, thereby protecting against I/R-induced increase in ROS and ferroptosis in the lung.

## Introduction

Medical conditions, such as transplantation, shock, cardiopulmonary bypass surgery, acute respiratory distress syndrome, and pulmonary embolism, can provoke ischemia/reperfusion (I/R) injury to the lung, which is associated with high mortality and deleteriously affects the success of lung transplantation (Kuratani et al. 1992; de Perrot et al. 2003; den Hengst et al. 2010). Lung I/R injury is an aseptic inflammation characterized by pulmonary edema and hypoxemia (Chen et al. 2017). The prevention of lung I/R injury is crucial in improving the survival of patients with severe cardiopulmonary disease or in need of lung transplantation (de Perrot et al. 2003; Hartwig and Davis 2004; Capuzzimati et al. 2022). Therefore, more research and strategies are needed to prevent and treat lung I/R injury (de Perrot et al. 2003; Chen and Date 2015).

Alveolar epithelial and vascular endothelial cells are injured in lung I/R injury, which is closely linked to the accumulation of inflammatory cells and production of reactive oxygen species (ROS) (Ferrari and Andrade 2015). Ferroptosis caused by the imbalance between the generation and degradation of intracellular lipid ROS plays an important pathological role in I/R injury (Chen and Fan et al. 2021). Inhibition of ferroptosis is considered to be an effective method to prevent I/R injury in several organs, including the lung (Pan et al. 2022; Wang et al. 2023).

Branched-chain amino acids (BCAA), such as leucine, isoleucine, and valine, are essential amino acids (Dimou et al. 2022). Impaired BCAA metabolism plays an important role in cardiac I/R injury, as ameliorating BCAA metabolism alleviates the I/R injury (Li et al. 2017; Lian et al. 2020). There are several explanations for this phenomenon. Impaired BCAA metabolism may cause tissue energy supply disorder (Li et al. 2017). The improvement of BCAA metabolism can reduce BCAA accumulation in organs; excessive accumulation of BCAA can produce toxic effects (Li et al. 2017, 2020). However, an increase in BCAA metabolites may have protective effects. BCAA metabolism can generate a variety of bioactive molecules, some of which have been reported to protect against organ I/R injury (Dong et al. 2016). Therefore, we suggest that enhanced BCAA metabolism can play a protective role against I/R injury through the production of protective metabolites.

L-β-aminoisobutyric acid (L-BAIBA) is a natural metabolite of valine, and exercise can increase circulating L-BAIBA concentrations (Kammoun and Febbraio 2014). Recent studies have shown that L-BAIBA can inhibit inflammation and induce fatty acid oxidation (Roberts et al. 2014; Jung et al. 2015). Moreover, L-BAIBA can also protect cells from ROS-induced damage (Kitase et al. 2018; Sawada et al. 2019). These studies suggest that L-BAIBA has protective effects in various organs. However, whether L-BAIBA can protect organs from I/R injury is unclear. The aim of our study is to verify the protective effect of L-BAIBA preconditioning and postconditioning on lung I/R injury and determine its protective mechanism.

## Materials and methods

### Animals

Eight-week-old C57BL/6J mice (20–30 g, SPF Biotechnology, Beijing, China) were obtained from the animal center of Army Medical University. The mice were kept in a Specific Pathogen-Free environment and fed a normal mouse diet. The mice can eat and drink freely, under a normal day and night cycle.

### Animal models

C57BL/6 mice were anesthetized with sodium pentobarbital (50 mg/kg, intraperitoneal injection), and placed on heating pads to maintain their body temperature. The right femoral arteries of the mice were cannulated with polyethylene tubing for arterial blood sampling. After ensuring the appropriate depth of anesthesia, the mice were intubated via tracheotomy and connected to a rodent ventilator (RWD Life Science, Shenzhen, China) with tidal volumes of 10 µl/g and a respiratory rate of 100 per minute. After thoracotomy, the hilum of the left lung of the I/R group was occluded with an atraumatic microclamp for 1 h to induce unilateral ischemia and then released for 1 h for reperfusion. The L-BAIBA treatment scheme is shown in **Supplemental Fig. **1A. To determine whether L-BAIBA preconditioning has a protective effect on I/R injury, the mice were first preconditioned by the gavage of L-BAIBA (150 mg/kg/day, 150 mg L-BAIBA dissolved in 8 ml dH_2_O) for 10 consecutive days. Then, the left lung was injured by I/R, as described above. For post-treatment, L-BAIBA (150 mg/kg, 150 mg L-BAIBA dissolved in 20 ml saline) was given by intraperitoneal injection immediately after I/R. Only the left lung was used in these experiments.

After the lung I/R injury, blood samples were immediately obtained for arterial blood gas analysis (Gem primer 3000, Instrumentation Laboratory) and enzyme-linked immunosorbent assay (ELISA). To obtain BALF, the mouse neck skin was cut, and the thyroid gland was separated from the trachea by blunt dissection. Then a surgical suture was placed behind the separated trachea. A small incision in the upper end of the trachea was made, into which was quickly inserted a catheter (0.7 mm diameter) connected to a small animal indwelling needle (22G). The catheter and the trachea were tied together with the surgical suture. A 1 ml syringe was loaded with normal saline and gently injected into the trachea; 0.5 ml of the saline was drained by gentle suction and repeated 3 times; 0.5 ml of BALF from each mouse was collected for subsequent experiments.

### Histological study

After performing the lung I/R injury, the mice were euthanized, and the lung tissues were rinsed with 30 ml pre-cooled PBS that were injected into the right ventricle to remove residual blood. The lung samples were stored in 4% paraformaldehyde for 24 h, then embedded in paraffin, sectioned, and placed on glass slides. After dewaxing and rehydrating with xylene and ethanol of different concentrations, the sections were processed by the following steps: stain with hematoxylin solution for 7 min, rinse with running water for 5 min, soak in 1% acid alcohol for 1 min, and counter-stain with eosin Y solution for 2 min. Finally, the plates were dehydrated and sealed with neutral resin. Smith pathological score was used to quantify the histological injury of the lungs, which included four indexes: pulmonary edema, alveolar and interstitial inflammation, alveolar and interstitial hemorrhage, and hyaline membrane formation (Smith et al. 1997).

### Wet/dry lung weight ratio

After the mice were sacrificed, the lungs were taken and weighed (wet weight), then placed in an oven at 80 °C for 24 h, and reweighed to determine the dry weight for the calculation of the wet/dry weight ratio (Luo et al. 2022).

### ELISA

To obtain the sera, the blood samples were collected from the right femoral artery by catheterization and allowed to coagulate at room temperature. Then the collected whole blood samples were quickly centrifuged at 1000 g for 5 min and the supernatants were collected. Care was taken to avoid hemolysis during the entire process. The BALF samples were centrifuged at 1000xg for 5 min and the supernatants were collected. The concentrations of IL-1β, IL-6, and TNF-α were measured by ELISA (Elabscience), according to the manufacturer’s instructions.

### Measuring the activities of MDA, MPO, SOD, and the ratio of GSH and GSSG

Malondialdehyde (MDA), glutathione (GSH), and glutathione disulfide (GSSG) activities were measured using kits purchased from Beyotime Biotechnology. Myeloperoxidase (MPO) and superoxide dismutase (SOD) activity assay kits were purchased from Jiancheng Biological Engineering Institute. Briefly, the obtained lung tissues were homogenized in the sample preparation solution and then the assays were performed, according to the manufacturer’s instructions.

### TUNEL staining

Paraffin sections of the lungs were dewaxed. Then, the sections were placed in an antigen repair solution and boiled in a water bath for 30 min. Thereafter, the boiled sections were sealed for 1 h after natural cooling. Then, the terminal deoxynucleotidyl transferase dUTP nick end labeling (TUNEL) staining solution, prepared according to the manufacturer, was added to the specimens and incubated at 37℃ for 30 min. Finally, the sections were dried and sealed with DAPI-containing immunostaining tablets. The images under a laser confocal microscope were obtained.

### DHE staining

Superoxide production in the lung was measured using the fluorescent dye dihydroethidium (DHE, Beyotime). For lung tissues, frozen sections of the lung were stained with DHE (10^− 5^ M) for 30 min in 37 °C. For cell samples, the cells were cultured on the cell slide. After the cell treatments, the cell slides were stained with DHE (10^-5^ M) for 30 min in 37 °C. Then, the cells were fixed with 4% paraformaldehyde for 15 min. After the tissues or slides were washed by PBS three times, the images were taken by a confocal microscope at an excitation wavelength of 490 nm and an emission wavelength of 590 nm. All sections were processed under the same conditions. The DHE fluorescence intensity was quantified by ImageJ (NIH, Bethesda).

### Western blotting

The total proteins were isolated from the lung tissues or cells. 20 mg of lung were lysed in 100 µl RIPA buffer, containing protease and phosphatase inhibitors cocktails (Solarbio), with Benchmark Beadblast tissue homogenizer (Benchmark Scientific). The protein concentration was measured with bovine serum albumin as a reference standard, according to BCA Protein Assay Kit (Beyotime Biotechnology). Nuclear and Cytoplasmic Protein Extraction Kit was purchased from Beyotime Biotechnology, and used according to the manufacturer’s instruction.

SDS-PAGE was performed using 8%, 10%, and 15% concentrations of SDS. The proteins separated by SDS-PAGE were transferred onto nitrocellulose filter membranes (Millipore Sigma). The nitrocellulose membranes were cropped according to molecular weights of the proteins and incubated with the appropriate antibodies. Image J (NIH, Bethesda, MD) was used to analyze the density of each band.

Antibodies used in this study were: anti-β-actin (1:500, Santa Cruz, SC-130,657), anti-H3 (1:3000, Cell Signaling Technologies, #4658), anti-GPX4 (1:500, Proteintech, 67763-1-Ig), anti-SLC7A11 (1:1000, Invitrogen, PA1-16893), anti-Nrf-2 (1:1000, Cell Signaling Technologies, #12721; 1:500, Proteintech, 80593-1-RR), anti-P53 (1:1000, Proteintech, 60283-2-Ig), anti-BAP1 (1:500, Affinity, AF7925), anti-PARP1 (1:1000, Proteintech, 22999-1-AP), anti-ATF3 (1:500, Santa Cruz, sc-81,189), anti-AMPK (1:1000, Cell Signaling Technologies, #2532), and anti-Phospho-AMPK (1:1000, Cell Signaling Technologies, #2535).

### A/R (anoxia/reoxygenation)-induced injury in A549 or BEAS-2B cells

A549 cells (Procell Life Science & Technology, Wuhan, China), which are human pulmonary alveolar cells, obtained from lungs with carcinoma, were cultured in DMEM high glucose medium (Gibco), containing 10% fetal bovine serum (FBS) and 1% penicillin/streptomycin (Beyotime). BEAS-2B cells, which are human bronchial epithelial cells (American Type Culture Collection), were cultured in bronchial epithelial cell basal medium, supplemented with BEGM (Lonza/Clonetics Corporation), containing 10% fetal bovine serum and 1% penicillin/streptomycin. The cells were cultured at 37℃ in 95% air and 5% CO_2_ atmosphere. After changing the incubation medium to serum-free medium, the cells were placed in an anoxic chamber with 5% CO_2_ and 95% N_2_ at 37℃ for 2 h, followed by reoxygenation in 95% air and 5% CO_2_ atmosphere for another 2 h. Exogenous L-BAIBA was added to the cells (100 µM-100 nM, final concentrations) 48 h before they were placed inside the anoxic chamber.

### Plasmid transfection and anti-oxidant response element (ARE) luciferase activity assays

DH5-competent E. coli was used for plasmid screening and amplification. A549 cells were seeded in 6-well plates and transfected with Lipofectamine 3000 at a cell confluency of approximately 50%, according to the standard experimental procedures provided in the manufacturer’s instructions. For the luciferase activity assay, the double-stranded oligonucleotide tandem repeats sequence spanning the Nrf-2 binding site 5-tgactcagca-3 was inserted into the restriction site of the pGL2 promoter plasmid to construct the ARE luciferase vector. Then, the cell lysate was mixed with the luciferase substrate solution, and the luciferase activity was measured with a luminometer (Xiao et al. 2018).

### CCK8 and LDH release assay

A549 or BEAS-2B cells were seeded into 96-well culture plates (Corning, Lowell, MA). The L-BAIBA preconditioning group was treated with different concentrations (10^− 7^ to 10^−^ ^4^ M) of L-BAIBA (Sigma) for 48 h. In addition, Nrf-2, AMPK, ERK, JNK, and PI3K inhibitors (Brusatol, Compound C, PD98059, SP600125, and LY294002, respectively) were added into the cell medium 30 min before preconditioning with L-BAIBA. RSL-3 and Erastin, two ferroptosis inducers, were added into the cell medium 24 h before hypoxia. Compound C, PD98059, SP600125, and LY294002 were purchased from Sigma-Aldrich. Brusatol, RSL-3 and Erastin were purchased from MCE.

A549 and BEAS-2B cells were allowed to grow to sub-confluence (70–80%). After the cells were subjected to A/R, CCK8 reagent (Bimake) and complete medium were added to the 96-well plates at a ratio of 1:9, then incubated at 37 °C for 1 h. Thereafter, the absorbance measured at 450 nm. LDH was measured by LDH cytotoxicity assay kit (Beyotime Biotechnology). 120 µl cell culture supernatants were collected and placed in a new 96-well plate. Then, 60 µl LDH detection working solution were added into each well, and incubated in the dark at 37℃ for 30 min. Finally, the absorbance was measured at 490 nm.

### Fe^2+^ staining

The cells were seeded in glass-bottomed Petri dishes (Thermo Scientific). After the cells were treated, 1 µl Fe^2+^ indicator storage solution (1 mM, dissolved with DMSO, Maokang Biotechnology) was added to 1 ml cell culture medium (final concentration 1 µM) and incubated for 30 min at 37℃ with 95% air and 5% CO2. After loading, the cells were washed with PBS. Fluorescent images were captured at 40 X total magnification using Olympus IX83 microscope (Olympus, Lake Success, NY).

### Immunofluorescence staining

The cells, cultured on glass slides, were fixed with 4% paraformaldehyde for 15 min after the treatments. Then, the glass slides with the cells were incubated with immunostaining blocking solution for 1 h at room temperature to prevent nonspecific antibody binding. Then, the cells in the glass slides were incubated with anti-Nrf-2 antibody (1:100) at 4℃ overnight. After washing with PBS 3 times (5 min each time), the cells in the glass slides were incubated with the secondary antibody, Cy3-labeled goat anti-rabbit IgG (1:200), at 37℃ for 30 min. After washing with PBS 3 times (5 min each time), the sections were stained with DAPI before being imaged under a confocal microscope.

### Statistical analysis

All data are represented as mean ± SD. The distribution of the experimental data was determined by Kolmogorov-Smirnov test. Data with non-normal distribution were analyzed by Kruskal-Wallis test. For the data with normal distribution and homogeneity of variance, one-way analysis of variance (ANOVA) was used to analyze the differences among multiple groups, and least significant difference (LSD) was used for two-group comparison. The Tamhane test was performed when assumptions of homogeneity of variance were questionable. SPSS 26 (IBM Corporation, Armonk, NY) was used for the statistical analyses. For survival analysis, the Log-rank (Mantel-Cox) test was used to determine the significance of differences in cumulative survival. GraphPad prism 8.0.2 software was used for the statistical analysis. A value of P < 0.05 was considered significant.

## Results

### L-BAIBA ameliorates the lung injury caused by ischemia-reperfusion

**Fig. 1:**
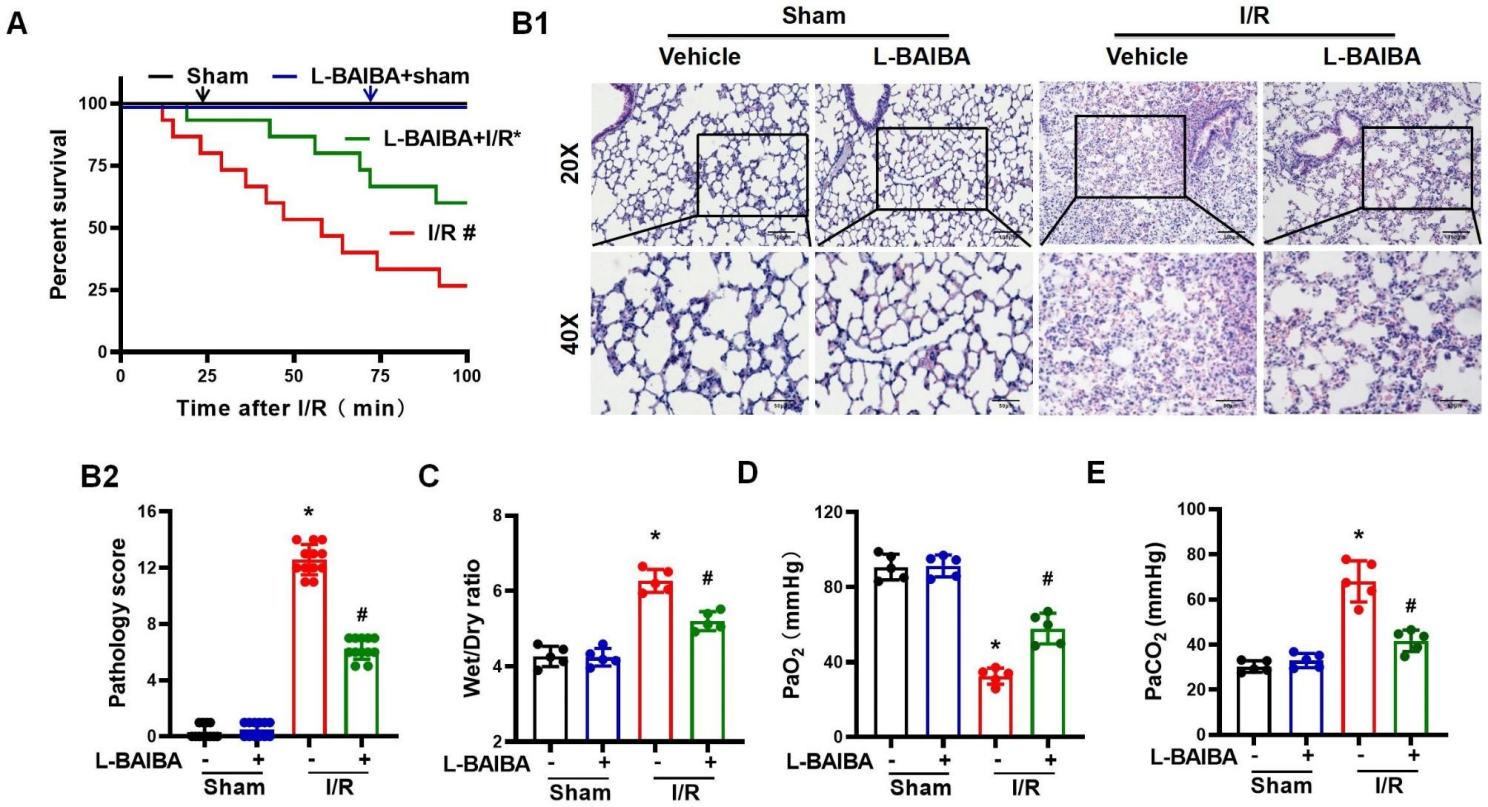
L-BAIBA preconditioning protects the lung from I/R injury **(A)**: Mouse survival rates after lung I/R injury with or without L-BAIBA treatment (n = 15, Log-rank (Mantel-Cox) test was used to analyze survival rate). **(B)**: Representative histopathological images of I/R-induced lung injury in mice with or without L-BAIBA treatment (n = 12, Kolmogorov-Smirnov test was used for normality test and Kruskal-Wallis test was used for statistical analysis). **(C)**: The degree of lung edema in mice was evaluated by the ratio of lung wet and dry weights (n = 5, Kolmogorov-Smirnov test was used for normality test. ANOVA was used for statistical analysis and LSD was used for post hoc analysis). (**D** and **E**): The arterial blood PaO_2_**(D)** and PaCO_2_**(E)** were used to evaluate the degree of hypoxemia (n = 5). Kolmogorov-Smirnov test was used for normality test. ANOVA was used for statistical analysis and LSD (**D**) or Tamhane test (**E**) was used for post hoc analysis). The values are presented as mean ± SD (*P < 0.05 vs. Sham, #P < 0.05 vs. I/R only)

To determine whether L-BAIBA preconditioning has a protective effect on lung I/R injury, we preconditioned the mice by the gavage of L-BAIBA (150 mg/kg/day in 200 µl dH_2_O) for 10 consecutive days. Then, the left lung was injured by I/R. We found that L-BAIBA preconditioning improved the survival of the mice subjected to left lung I/R injury (Fig. 1A). The pathological Smith scores (Smith et al. 1997) and pulmonary edema were significantly improved in mice with I/R injury treated with L-BAIBA compared with mice subjected to I/R injury only (Fig. 1B and C). Moreover, pulmonary oxygenation, another marker of lung I/R injury, measured by the partial pressure of oxygen (PaO_2_) and partial pressure of carbon dioxide (PaCO_2_), was improved by L-BAIBA treatment, increasing the PaO_2_ (Fig. 1D) and decreasing the PaCO_2_ (Fig. 1E) towards the sham-treated values.

**Fig. 2:**
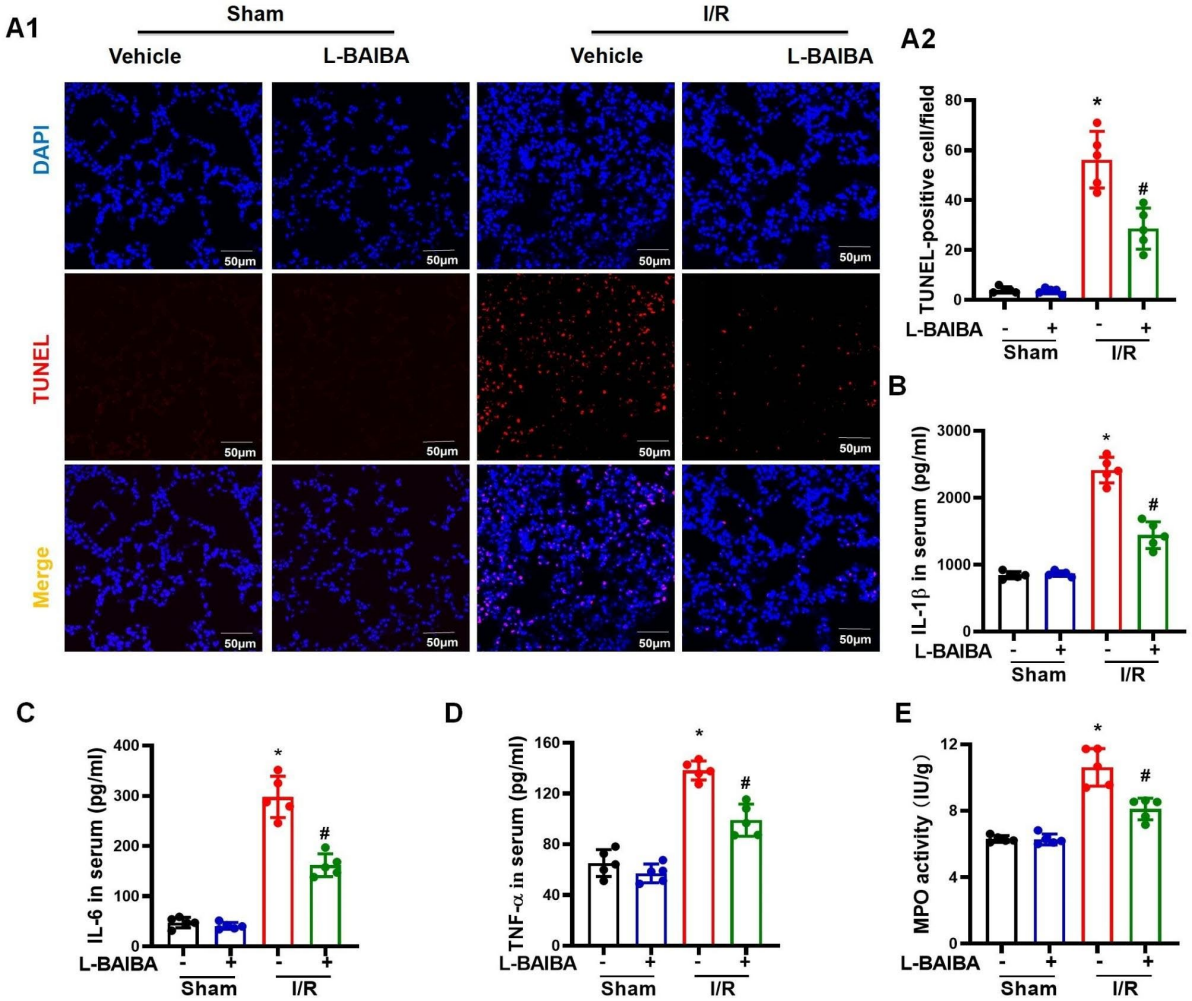
L-BAIBA ameliorates the apoptosis and inflammatory response in the lung subjected to I/R injury **(A)**: Results of TUNEL staining showed that L-BAIBA inhibited I/R-induced cell apoptosis. The experiment was repeated 5 times, and representative images are shown (n = 5). (**B**-**D**): Serum concentrations of interleukin-1β (IL-1β) **(B)**, IL-6 **(C)**, and TNF-α **(D)** were measured by ELISA (n = 5). (**E**): MPO activity in lungs was measured by commercially available kits (n = 5). Kolmogorov-Smirnov test was used for normality test. ANOVA was used for statistical analysis and Tamhane test (**A**, **B**, **C**, and **E**) or LSD (**D**) was used for post hoc analysis). The values are presented as mean ± SD (*P < 0.05 vs. Sham, #P < 0.05 vs. I/R only)

Apoptosis is another characteristic of lung I/R injury. TUNEL staining showed that L-BAIBA treatment reduced the number of TUNEL-positive cells in the lungs with I/R injury (Fig. 2A**)**. L-BAIBA treatment also reduced the concentrations of the pro-inflammatory cytokines IL-1β, IL-6, and TNF-α in serum (Fig. 2B - D) and BALF (**Figures **S2**A-S2C**) in mice with lung I/R injury. L-BAIBA preconditioning also reduced the activity of MPO, another biomarker of inflammation, in lung with I/R injury (Fig. 2E**)**. In addition to studying the effect of pre-treatment with L-BAIBA, we also studied the effect of L-BAIBA treatment after the I/R injury. We found that post-treatment with L-BAIBA also protected the lung from I/R injury **(Figures **S3**A-3D)**, although the protection was less than pre-treatment. These results indicate that L-BAIBA has beneficial effects before and after the occurrence of lung I/R injury.

### L-BAIBA ameliorates the ferroptosis induced by ischemia-reperfusion

**Fig. 3:**
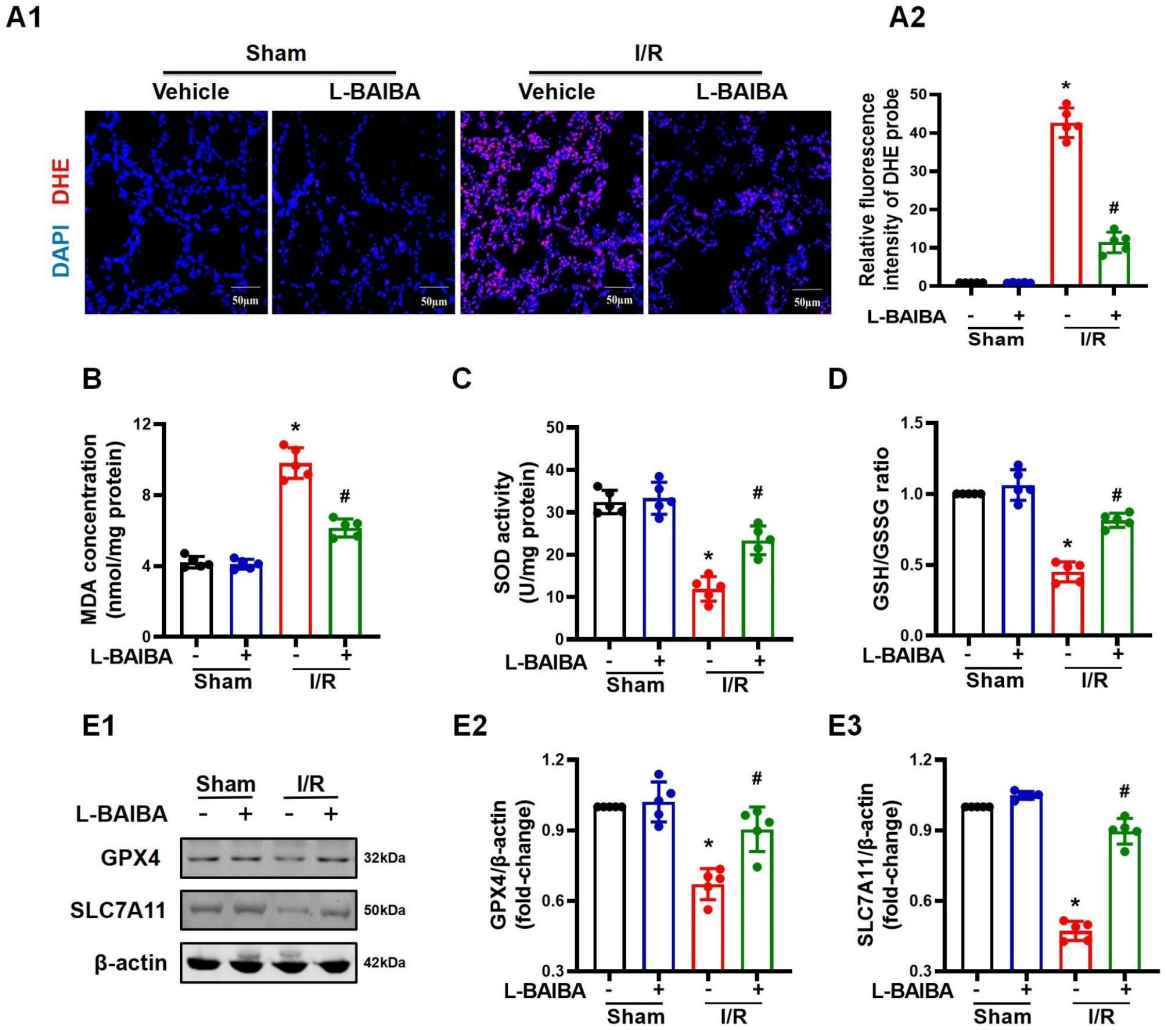
L-BAIBA protects the lung against ferroptosis induced by I/R injury **(A)**: The level of reactive oxygen species (ROS) in the lung was evaluated by dihydroethidium (DHE) staining (n = 5). Representative images are shown. The fluorescence intensity was analyzed by Image J (n = 5). **(B)**: The concentrations of MDA in the lungs were measured (n = 5). **(C)**: The SOD activity in the lungs was measured by commercially available kits (n = 5). **(D)**: The ratio of GSH to GSSG was used to measure the oxidative stress level (n = 5). **(E)**: Western blot was used to detect changes in the protein expression levels of GPX4 and SLC7A11 in the lung injured by I/R with or without L-BAIBA treatment (n = 5). Kolmogorov-Smirnov test was used for normality test. ANOVA was used for statistical analysis and Tamhane test (**A**, **B**, **D**, and **E3**) or LSD (**C** and **E2**) was used for post hoc analysis. The values are presented as mean ± SD (*P < 0.05 vs. Sham, #P < 0.05 vs. I/R only)

**Fig. 4:**
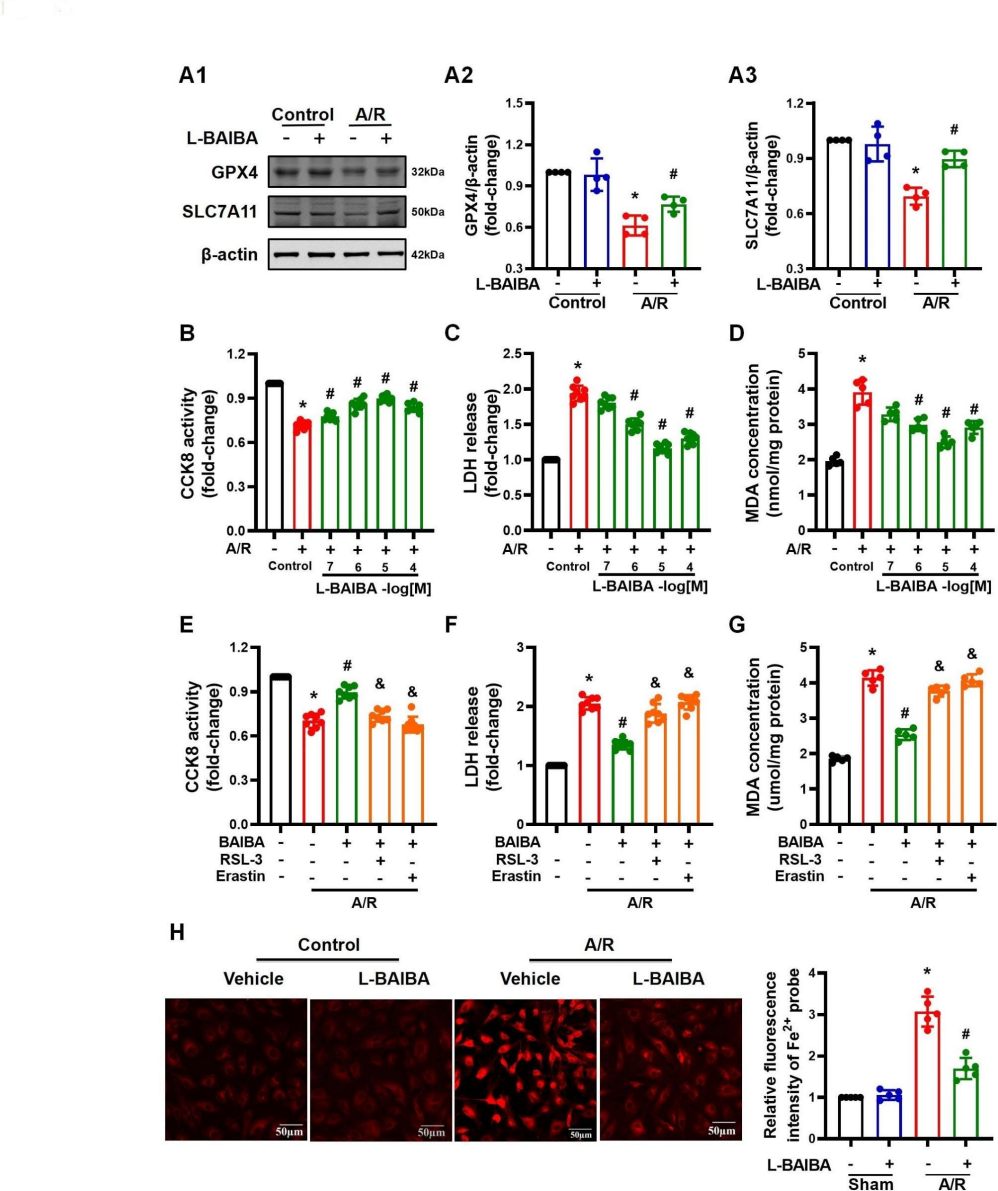
L-BAIBA protects A549 cells from A/R injury by inhibiting ferroptosis **(A)**: Western blot was used to detect the protein expressions of GPX4 and SLC7A11 in A549 cells before or after A/R injury with or without L-BAIBA treatment (n = 4). **(B** and **C)**: Effects of L-BAIBA on A549 cells injured by A/R were determined by measuring CCK8 activity and LDH release. A549 cells were treated with different concentrations of L-BAIBA (0.1–100 µM) (n = 8). **(D)**: MDA concentration was to detect lipid peroxidation in A549 cells (n = 5). **(E** and **F)**: The effects of the ferroptosis inducers RSL-3 and erastin on the activity of A/R-injured cells were determined by CCK8 activity (n = 8) and the release of LDH (n = 8). Kolmogorov-Smirnov test was used for normality test and Kruskal-Wallis test was used for statistical analysis. **(G)**: The effect of ferroptosis inducers RSL-3 and erastin on lipid peroxidation after A/R injury was detected by measuring MDA concentrations (n = 5). **(H)**: Images of Fe^2+^ probe staining. The experiment was repeated 5 times, and a representative image is shown. Average fluorescence intensity was analyzed by Image J. The values are presented as mean ± SD (*P < 0.05 vs. Control, #P < 0.05 vs. A/R only, &P < 0.05 vs. L-BAIBA + A/R). Kolmogorov-Smirnov test was used for normality test. ANOVA was used for statistical analysis and post hoc analysis was performed by using Tamhane test (**A3**, **B**, **C**, **D**, and **F**) or LSD (**A2**, **G**, and **H**)

Ferroptosis is a form of programmed cell death caused by iron-dependent accumulation of lipid peroxides. Lipid oxidation is characteristic of ferroptosis and the impairment of the antioxidant system is the pathological basis of ferroptosis (Chen and Fan et al. 2021). L-BAIBA is an effective antioxidant, which can positively regulate the expression of various antioxidant molecules (Sawada et al. 2019). However, whether the antioxidant effect of L-BAIBA can inhibit ferroptosis is still unclear. In this study, we found that L-BAIBA preconditioning ameliorated the oxidative stress in the lung injured by I/R, proved by the decrease in DHE fluorescence and MDA level, and increase in SOD activity and the GSH/GSSG ratio (Fig. 3A - D). Additional studies revealed that GPX4 and SLC7A11, the primary defense proteins against ferroptosis (Chen and Yu et al. 2021), were up-regulated in I/R-injured lungs of mice that were pretreated with L-BAIBA (Fig. 3E). These results were verified in vitro. In A549 cells subjected to A/R, GPX4 and SLC7A11 protein expressions were decreased but were up-regulated by L-BAIBA (Fig. 4A). Meanwhile, L-BAIBA also effectively protected the cells injured by A/R, as determined by the increase in CCK8 activity, and decrease in LDH release and MDA concentration (Fig. 4B - D). It should be noted that 10^− 7^ to 10^− 4^ M concentrations of L-BAIBA were not toxic to A549 cells (**Figure **S4**A**). We further tested the role of L-BAIBA in the regulation of ferroptosis by performing blocking experiments. We found that two inducers of ferroptosis, RSL-3 (10 µM) and erastin (10 µM), were able eliminate the protective effect of L-BAIBA on cell damage induced by A/R, demonstrated by their ability to reverse the effects of L-BAIBA on CCK8 activity, LDH release, and MDA concentration (Fig. 4E - G). Therefore, the above results confirmed that the protective effect of L-BAIBA may be achieved by inhibiting ferroptosis. Indeed, Fe^2+^ staining in A549 cells that was markedly increased after A/R exposure was markedly reduced by L-BAIBA (Fig. 4H). These results show that L-BAIBA may protect against lung I/R injury by up-regulating the expressions of GPX4/SLC7A11 and subsequently inhibiting ferroptosis.

### L-BAIBA inhibits ferroptosis through the Nrf-2 signaling pathway

**Fig. 5:**
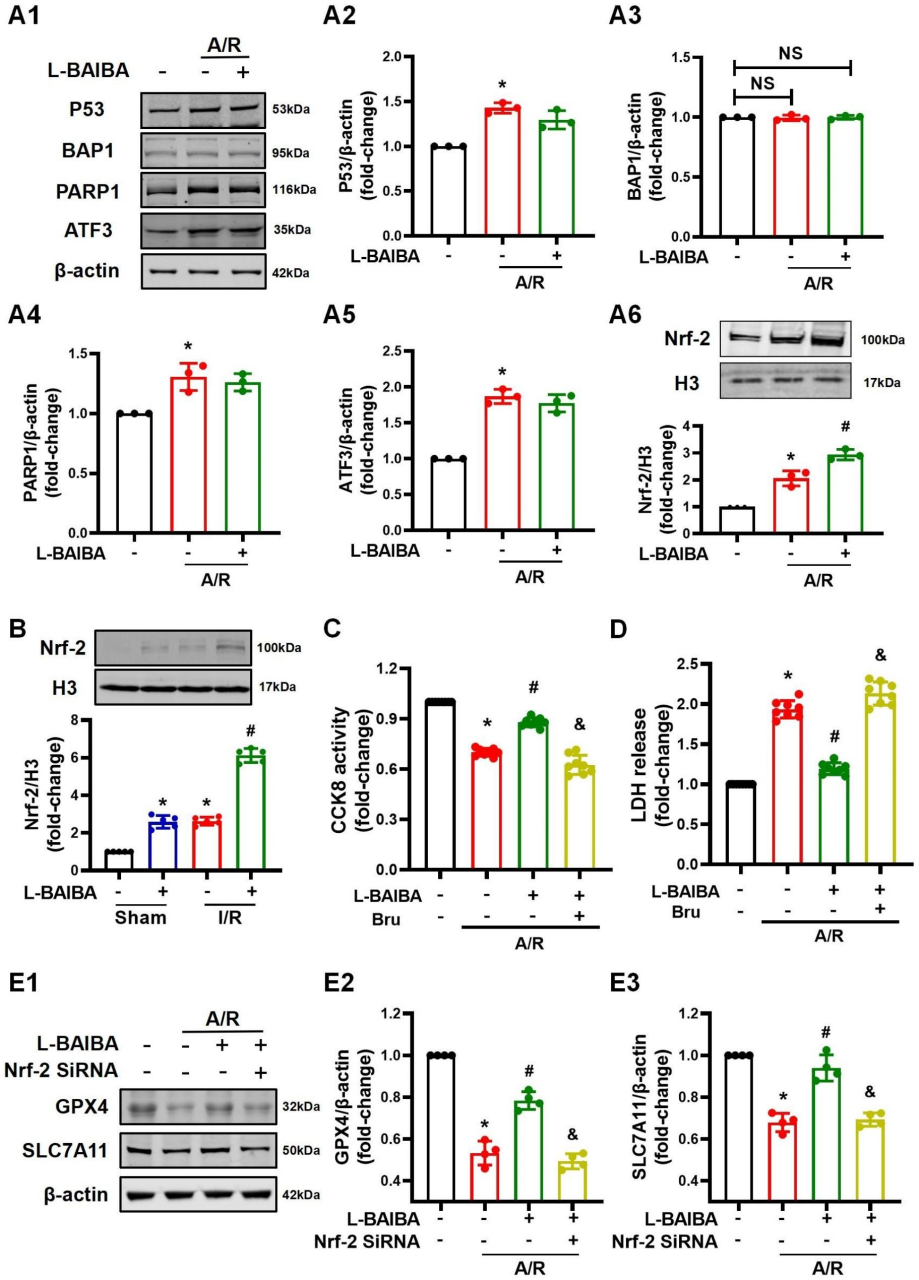
L-BAIBA inhibits ferroptosis through the Nrf-2 signaling pathway in A549 cells **(A)**: Western blot was used to detect the changes in the protein expressions of several anti-ferroptosis signaling proteins, such a P53, BAP1, PARP1, ATF3, and Nrf-2 (n = 3). **(B)**: The protein levels of Nrf-2 in the nuclear extracts were analyzed by western blot lungs with or without I/R and with or without L-BAIBA treatment (n = 5). **(C** and **D)**: The role of Nrf-2 signaling pathway on the protective effect of L-BAIBA was confirmed by blocking experiments; brusatol was used as the inhibitor of Nrf-2. The effects were determined by measuring CCK8 activity and LDH release (n = 8). **(E)**: Western blot was used to detect changes in the protein expressions of GPX4 and SLC7A11 in A549 cells with or without interfering the expression of Nrf-2 (n = 4). The values are presented as mean ± SD (*P < 0.05 vs. Control or Sham, #P < 0.05 vs. A/R or I/R only, &P < 0.05 vs. L-BAIBA + A/R). Kolmogorov-Smirnov test was used for normality test. ANOVA was used for statistical analysis and post hoc analysis was performed by using LSD (**A2**, **A5**, **A6**, and **E**) or Tamhane test (**A3**, **A4**, **B**, **C**, and **D**)

We further explored the possible molecular mechanism by which L-BAIBA regulates the expressions of GPX4 and SLC7A11. Using the information from previous reports, several signaling molecules that could regulate GPX4 and SLC7A11 were screened (Hong et al. 2021; Wang et al. 2021; Fu et al. 2022; Ye et al. 2022; Zeng et al. 2022) (Fig. 5A). The results showed that L-BAIBA stimulated only the expression of Nrf-2 (Fig. 5A and B**)**, which suggested that Nrf-2 may be a signaling pathway in the protective effect of L-BAIBA against I/R and A/R. Therefore, further experiments were performed. The regulatory effect of BAIBA on Nrf-2 signaling pathway was confirmed in the lung because L-BAIBA induced the nuclear translocation of Nrf-2 (Fig. 5B). The A549 cells experiments showed that L-BAIBA increased the level of Nrf-2 in the nucleus and the transcriptional activity of ARE in a concentration-dependent manner (**Figures **S5**A** and **S5B**). Moreover, brusatol (20 µM), an inhibitor of Nrf-2, blocked the protective effect of L-BAIBA on A549 cells A/R injury, measured by CCK8 activity and LDH release (Fig. 5 C and 5D). Finally, interfering with the expression of Nrf2 by transfecting Nrf-2 siRNA eliminated the ability of L-BAIBA to increase the protein expressions of GPX4 and SLC7A11 (Fig. 5E), which are involved in inhibitory effect of Nrf-2 on ferroptosis (vide infra). These pieces of evidence strongly support the notion that L-BAIBA inhibits ferroptosis by stimulating the Nrf-2 signaling pathway.

### L-BAIBA, by activating AMPK, induces Nrf-2 nuclear translocation

**Fig. 6:**
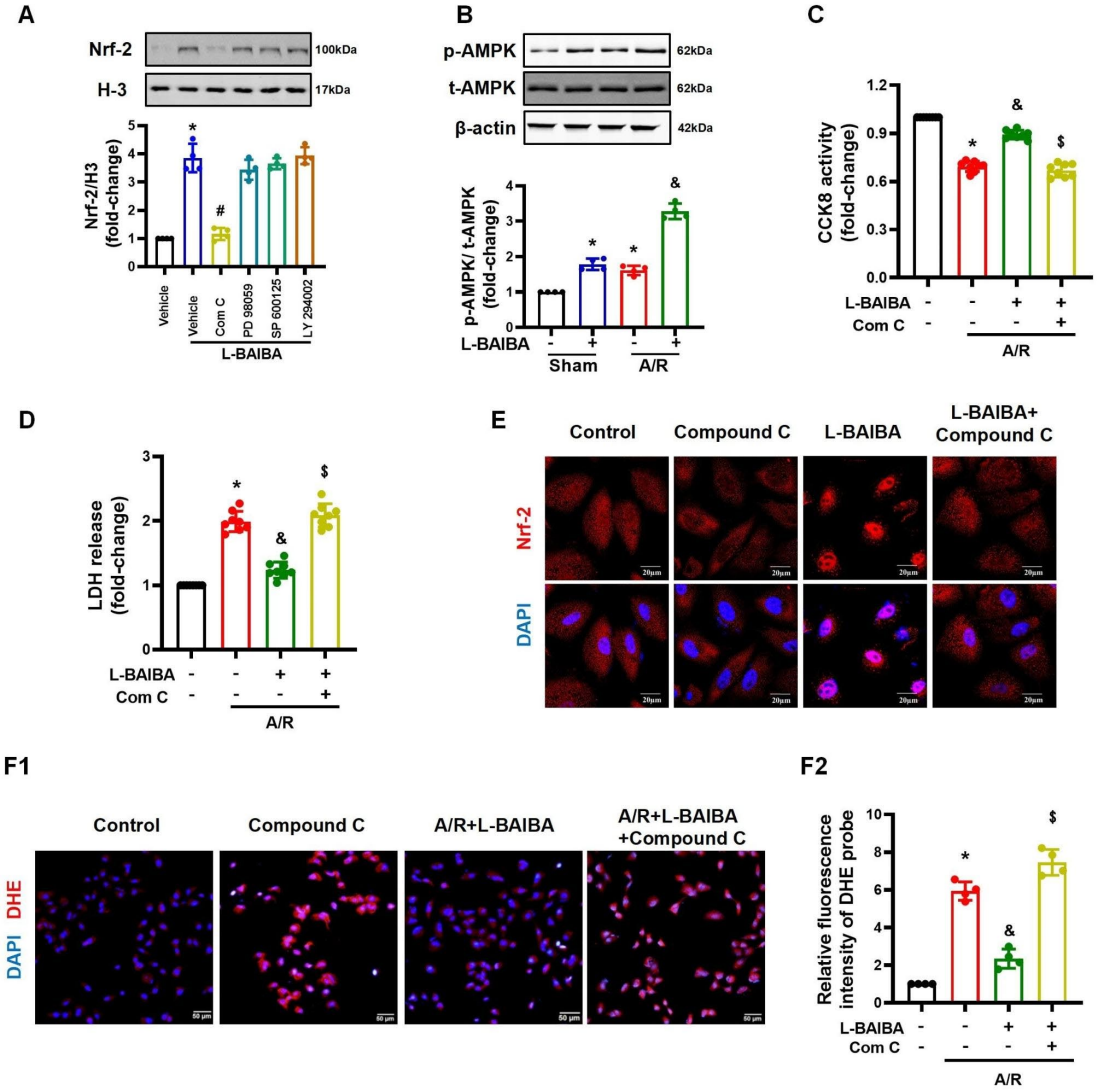
L-BAIBA promotes Nrf-2 nuclear translocation by activating the AMPK signaling pathway **(A)**: Role of AMPK in the regulatory effects of L-BAIBA on the nuclear translocation of Nrf-2 in A549 cells. A549 cells were treated with L-BAIBA in the presence of AMPK inhibitor compound C (10 µM), ERK inhibitor PD98059 (10 µM), Jun inhibitor SP600125 (20 µM) or PI3K inhibitor LY294002 (25 µM) (n = 4). **(B)**: The activating effect of L-BAIBA on AMPK signaling pathway was studied in A549 cells subjected to A/R (n = 4). **(C** and **D)**: The protective effect of L-BAIBA on A/R injury in A549 cells could be eliminated by compound C, as determined by measuring CCK8 activity and LDH release (n = 8). **(E)**: The regulatory effect of L-BAIBA on Nrf-2 nuclear translocation in A549 cells is shown by immunofluorescence staining. **(F)**: The levels of ROS in A549 cells were detected by DHE staining; the average fluorescence intensities are shown (n = 4). The values are presented as mean ± SD (*P < 0.05 vs. Control or Sham, #P < 0.05 vs. BAIBA only, &P < 0.05 vs. A/R only, $P < 0.05 vs. L-BAIBA + A/R). Kolmogorov-Smirnov test was used for normality test. ANOVA was used for statistical analysis and post hoc analysis was performed by using LSD (**A**) or Tamhane test (**B**, **C**, **D**, and **F**)

We further studied the underlying mechanisms by which L-BAIBA regulated Nrf-2 signaling pathway in A549 cells. It has been reported that some signaling pathways, such as AMPK, ERK, JNK, and PI3K/Akt, could regulate the nuclear translocation of Nrf-2 (Prieto et al. 2010; Huang et al. 2020; Xingyue et al. 2021). Therefore, we studied these signaling pathways by the administration of specific inhibitors of these pathways, including compound C (10 µM), an inhibitor of the AMPK pathway, PD98059 (10 µM), an inhibitor of the MEK/ERK pathway, SP600125 (20 µM), an inhibitor of autophagy and activator of apoptosis, and LY294002 (25 µM), an inhibitor of PI3K activity. The results showed that only compound C, an inhibitor of AMPK signaling pathway, was able to block the nuclear translocation of Nrf-2 induced by L-BAIBA (Fig. 6A). Further experiments showed that L-BAIBA was able to promote the phosphorylation of AMPK at Thr172 (Fig. 6B). However, in the presence of compound C, the protective effect of L-BAIBA on A549 cells, measured by an increase in CCK activity and decrease in LDH release, was blocked (Fig. 6 C and 6D). The inhibitory effect of compound C on Nrf-2 nuclear translocation induced by L-BAIBA was also detected by immunofluorescence staining (Fig. 6E). Finally, compound C also abolished the protective effect of L-BAIBA on A/R-induced oxidative stress in vitro in A549 cells (Fig. 6F). These studies confirmed that L-BAIBA promotes Nrf-2 signaling by activating the AMPK pathway. Some of the above-mentioned results from A549 cells, including cell activity, were confirmed in BEAS-2B cells, a non-malignant human lung bronchus epithelial cell line (**Figures **S6**A-S6D**). These results suggest that L-BAIBA can prevent ferroptosis in BEAS-2B cells through an AMPK-mediated pathway.

### The protective effect of L-BAIBA on lung I/R injury is abolished by blocking the AMPK signaling pathway

**Fig. 7:**
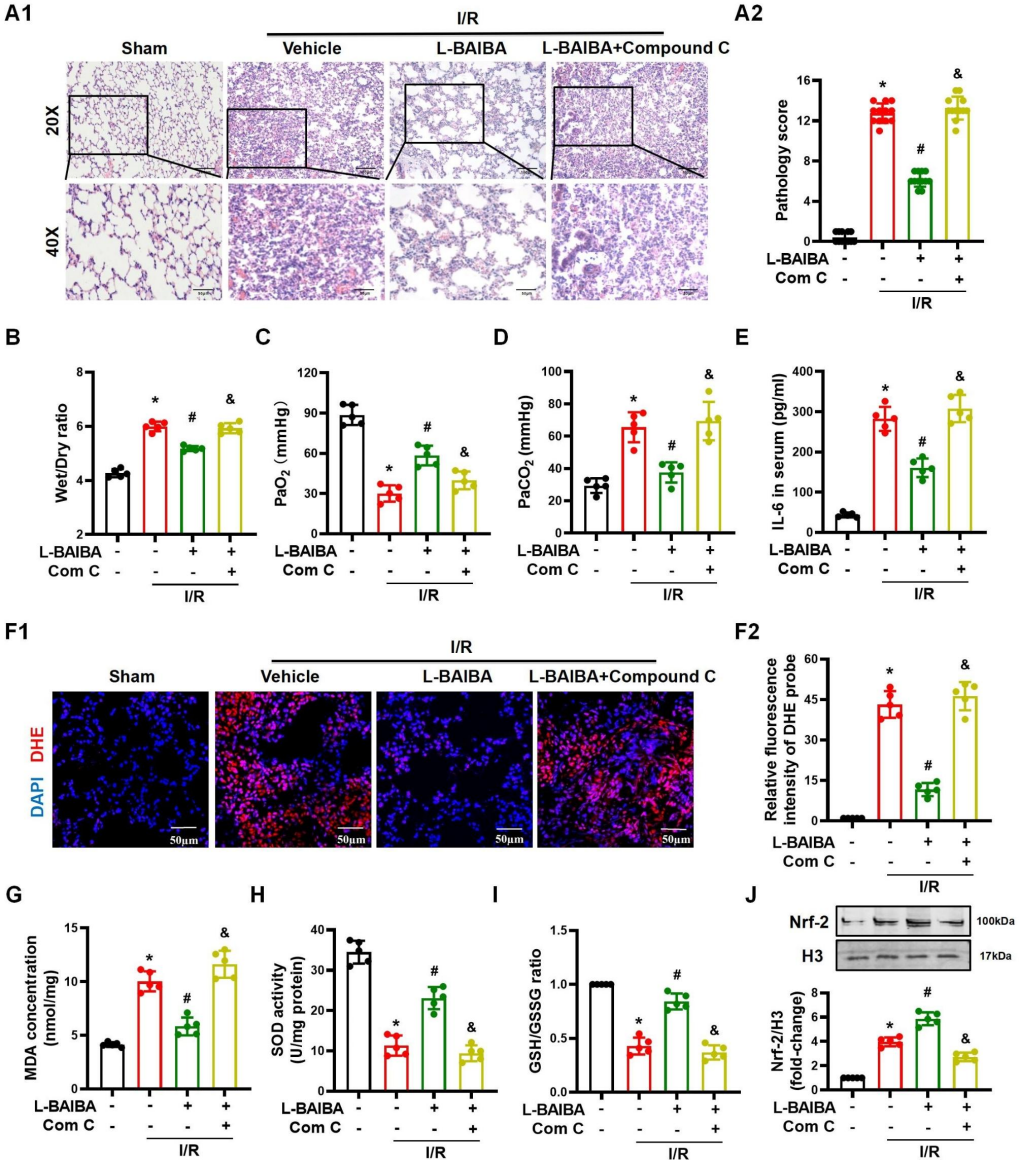
Inhibiting the AMPK signaling pathway eliminates the protective effects of L-BAIBA on lung I/R injury **(A)**: H&E staining shows the pathological changes in the lung injured by I/R (n = 12, Kolmogorov-Smirnov test was used for normality test and Kruskal-Wallis test was used for statistical analysis). **(B)**: Wet/dry weight ratio in lungs (n = 5). **(C** and **D)**: The arterial blood PaO_2_**(C)** and PaCO_2_**(D)** were measured to evaluate pulmonary oxygenation during IR injury (n = 5). (**E**): The concentrations of IL-6 in serum were measured by ELISA (n = 5). **(F)**: The production of ROS in lungs was measured by DHE fluorescence intensity, analyzed by Image J (n = 5). **(G)**: MDA concentration in the lungs was measured (n = 5). **(H)**: The SOD activity in the lungs was measured (n = 5). **(I)**: The ratio of GSH to GSSG was calculated (n = 5). **(J)**: The protein level of Nrf-2 in the nuclear extracts from mice was analyzed by western blot (n = 5). The values are presented as mean ± SD (*P < 0.05 vs. Sham, #P < 0.05 vs. I/R only, &P < 0.05 vs. L-BAIBA + I/R). Kolmogorov-Smirnov test was used for normality test. ANOVA was used for statistical analysis and post hoc analysis was performed by using Tamhane test (**F**, **G**, and **J**) or LSD (**B**, **C**, **D**, **E**, **H**, and **I**)

In the presence of compound C (10 mg/kg/day, dissolved in 200 µl normal saline, intravenous injection), the protective effects of L-BAIBA on lung I/R injury in C57BL/6 mice were eliminated. Treatment with compound C also prevented the ability of L-BAIBA to decrease the pathological damage in the lung caused by I/R injury (Fig. 7A). Compound C also prevented the ability of L-BAIBA to decrease the wet weight to dry weight ratio, an indicator of lung edema (Fig. 7B). The arterial blood gas analyses also showed that compound C was able to prevent the increase in PaO_2_ and decrease in PaCO_2_ caused by L-BAIBA in mice with lung I/R injury (Fig. 7 C and 7D**)**. Moreover, compound C also had a deleterious effect on the concentrations of IL-6 in serum and BALF because it eliminated the ameliorative effect of L-BAIBA in lung I/R injury (Fig. 7E and S7A). The administration of compound C also prevented the protective effects of L-BAIBA on the oxidative stress in lung I/R injured mice, confirmed by the persistence of the increased DHE staining of the lung, increased MDA concentration, and decreased SOD activity and GSH and GSSG ratio (Fig. 7F - I). Finally, compound C was also able to block the nuclear translocation of Nrf-2 induced by L-BAIBA in lungs with I/R injury in vivo (Fig. 7J), which is consistent with the results of the in vitro experiments (Fig. 6A and E). These results confirm that the AMPK signaling pathway is essential for the protective effect of L-BAIBA on lung I/R injury.

## Discussion

Organ I/R injury is thought to be accompanied by a defect in BCAA catabolism, which can be protected by enhancing normal BCAA metabolism (Lian et al. 2020). We hypothesize that the protective effect of BCAA may be related to an increase in BCAA active metabolites. L-BAIBA, a metabolite of valine and thymine, is an important muscle-derived metabolic factor (Kammoun and Febbraio 2014). Previous studies have found that L-BAIBA increases energy consumption by activating the β-oxidation of liver fatty acids, triggering the browning of white adipose tissue (Roberts et al. 2014). L-BAIBA also decreases insulin resistance and induces the expression of genes related to fatty acid oxidation through AMPK signaling (Jung et al. 2015). Because of these beneficial effects, L-BAIBA has become an attractive drug in the treatment of metabolic diseases. L-BAIBA has also been found to protect against multiple organ injury, which are related to its anti-oxidative and anti-inflammatory effects and alleviation of endoplasmic reticulum stress. Indeed, the circulating concentrations of L-BAIBA are negatively associated with cardio-metabolic risk factors (Roberts et al. 2014; Shi et al. 2016; Kitase et al. 2018). It is also worthwhile noting that L-BAIBA has favorable pharmacological properties, such as stability and convenient storage. L-BAIBA, being a promising therapeutic agent, has been the subject of toxicological and pharmacokinetic studies (Krieger et al. 2022; Shanmugasundaram et al. 2022).

In our study, we found that pretreating mice with L-BAIBA before lung I/R injury alleviated the cellular injury and edema, reduced the inflammatory cell infiltration, decreased apoptosis and oxidative stress, and improved pulmonary oxygenation. In addition, L-BAIBA reduced the serum and BALF levels of inflammatory cytokines, such as IL-1β, IL-6, and TNF-α. These results confirmed the protective effect of L-BAIBA on lung I/R injury.

Excessive oxidative stress can directly cause cytotoxic damage by impairing mitochondrial function or aggravating the inflammatory response (Orrenius et al. 2007). Ferroptosis, a type of cell death caused by oxidative stress, acts as a hub, linking oxidative stress, inflammation, and cell death (Yu et al. 2021a, b). Intracellular iron ions interact with fatty acids to produce lipid ROS. When the GSH antioxidant system is not repaired in time, lipid peroxidation and ferroptosis occur. Therefore, dysfunction of lipid peroxide clearance, the presence of redox-active Fe^2+^, and the oxidation of phospholipids containing polyunsaturated fatty acids are considered to be the three essential features of ferroptosis (Yu Yu et al. 2021a, b). Ferroptosis is thought to play an important role in lung I/R injury (Ferrari and Andrade 2015). Recent studies have found that ferroptosis is also closely related to the occurrence and development of many lung diseases (Yu and Jia Yu et al. 2021a, b; Li et al. 2022). There is no doubt that inhibition of oxidative stress and ferroptosis could be an effective strategy to prevent lung I/R injury.

In this study, increased MDA levels and decreased SOD activities, accompanied with decreased ratio of GSH and GSSG were found in the lung injured by I/R. However, preconditioning with L-BAIBA reduced oxidative stress, mitigated the increase in intracellular Fe^2+^ levels and almost completely restored the decreased expression of GPX4 and SLC7A11, which confirmed the anti-oxidant and anti-ferroptosis effects of L-BAIBA in lung I/R injury.

Further experiments showed that transcription factor Nrf-2 was involved in the protective effects of L-BAIBA. Nrf-2 regulates the expression of many genes involved in organ and cell defense, including antioxidants, detoxification enzymes, and drug transporters, among others. The transcription of these protective genes enables the cells to maintain normal redox equilibrium and eliminate proteins damaged by oxidative stress. Nrf-2 signaling pathway has also been shown to have a strong anti-ferroptosis effect (Wang et al. 2022; Zhang et al. 2022). Mounting pieces of evidence have shown that the up-regulation of Nrf-2 can protect various organs from I/R injury (Ucar et al. 2021). In this study, we found that L-BAIBA induced the nuclear translocation of Nrf-2. Inhibition of Nrf-2 activity or expression eliminated the protective effects of L-BAIBA on oxidative stress. The ability of L-BAIBA to upregulate the expression of GPX4 and SLC7A11 was also eliminated by interfering with Nrf-2 expression. These results further confirmed the contribution of Nrf-2 in the L-BAIBA-mediated protection of lung I/R injury.

AMPK signaling pathway is an important regulator of energy metabolism and has been shown to play a protective role against I/R injury (Ding et al. 2020). AMPK signaling also acts as a regulator of intracellular redox equilibrium, and the activation of AMPK can induce the translocation of Nrf-2 into the nucleus (Wang et al. 2019, 2022; Zhang et al. 2022). In our study, by screening a variety of signaling pathways which have been reported to regulate Nrf-2 signaling, we found that only the inhibition of AMPK activity could prevent the ability of L-BAIBA to induce the nuclear translocation of Nrf-2. In order to determine the clinical significance of the beneficial effect of L-BAIBA on cell metabolism, we treated the lung I/R injured mice with both compound C and L-BAIBA. Results showed that compound C eliminated the protective effect of L-BAIBA. Therefore, these results indicated that the protective effect of L-BAIBA on lung I/R injury depended on the AMPK signaling pathway.

We realize that there are some limitations of the present study. For example, the localization of L-BAIBA was not confirmed by tracer method and the side-effects of L-BAIBA in the in vivo studies were not monitored and should be studied in live animals in the future.

## Conclusion

L-BAIBA protects against lung I/R injury by inhibition of ferroptosis, which depended on the up-regulation of the expression of GPX4 and SLC7A11 by activating the AMPK/Nrf-2 signaling pathway. L-BAIBA may be a therapeutic drug for the prevention and treatment of lung I/R injury.

### Electronic supplementary material

Additional file of β-aminoisobutyrics Acid, a Metabolite of BCAA, Activates the AMPK/Nrf-2 Pathway to Prevent Ferroptosis and Ameliorates Lung Ischemia-Reperfusion Injury. Below is the link to the electronic supplementary material.

Supplementary Material 1

Supplementary Material 2

Supplementary Material 3

Supplementary Material 4

Supplementary Material 5

Supplementary Material 6

Supplementary Material 7

Supplementary Material 8

Supplementary Material 9

Supplementary Material 10

## Data Availability

The data used to support the conclusion of this study are included in the article.

## References

[CR1] Capuzzimati M, Hough O, Liu M (2022). Cell death and ischemia-reperfusion injury in lung transplantation. J Heart Lung Transplant.

[CR2] Chen F, Date H (2015). Update on ischemia-reperfusion injury in lung transplantation. Curr Opin Organ Transplant.

[CR3] Chen K, Xu Z, Liu Y (2017). Irisin protects mitochondria function during pulmonary ischemia/reperfusion injury. Sci Transl Med.

[CR5] Chen Y, Wang S (2021). Ferroptosis: a Novel Therapeutic Target for Ischemia-Reperfusion Injury. Front Cell Dev Biol.

[CR4] Chen X, Yu C, Kang R (2021). Cellular degradation systems in ferroptosis. Cell Death Differ.

[CR6] De Perrot M, Liu M, Waddell TK (2003). Ischemia-reperfusion-induced lung injury. Am J Respir Crit Care Med.

[CR7] Den Hengst WA, Gielis JF, Lin JY (2010). Lung ischemia-reperfusion injury: a molecular and clinical view on a complex pathophysiological process. Am J Physiol Heart Circ Physiol.

[CR8] Dimou A, Tsimihodimos V, Bairaktari E. The critical role of the branched chain amino acids (BCAAs) catabolism-regulating enzymes, branched-chain aminotransferase (BCAT) and branched-chain alpha-keto acid dehydrogenase (BCKD), in human pathophysiology. Int J Mol Sci 2022; 23 (7). 10.3390/ijms23074022 PMC8999875 35409380

[CR9] Ding R, Wu W, Sun Z (2020). AMP-activated protein kinase: an attractive therapeutic target for ischemia-reperfusion injury. Eur J Pharmacol.

[CR10] Dong W, Zhou M, Dong M (2016). Keto acid metabolites of branched-chain amino acids inhibit oxidative stress-induced necrosis and attenuate myocardial ischemia-reperfusion injury. J Mol Cell Cardiol.

[CR11] Ferrari RS, Andrade CF. Oxidative Stress and Lung Ischemia-Reperfusion Injury. Oxid Med Cell Longev 2015; 2015:590987. 10.1155/2015/590987 PMC4487720 26161240

[CR12] Fu C, Wu Y, Liu S (2022). Rehmannioside A improves cognitive impairment and alleviates ferroptosis via activating PI3K/AKT/Nrf2 and SLC7A11/GPX4 signaling pathway after ischemia. J Ethnopharmacol.

[CR13] Hartwig MG, Davis RD (2004). Surgical considerations in lung transplantation: transplant operation and early postoperative management. Respir Care Clin N Am.

[CR14] Hong T, Lei G, Chen X (2021). PARP inhibition promotes ferroptosis via repressing SLC7A11 and synergizes with ferroptosis inducers in BRCA-proficient ovarian cancer. Redox Biol.

[CR15] Huang XT, Liu W, Zhou Y (2020). Galectin-1 ameliorates lipopolysaccharide-induced acute lung injury via AMPK-Nrf2 pathway in mice. Free Radic Biol Med.

[CR16] Jung TW, Hwang HJ, Hong HC (2015). BAIBA attenuates insulin resistance and inflammation induced by palmitate or a high fat diet via an AMPK-PPARdelta-dependent pathway in mice. Diabetologia.

[CR17] Kammoun HL, Febbraio MA (2014). Come on BAIBA light my fire. Cell Metab.

[CR18] Kitase Y, Vallejo JA, Gutheil W (2018). beta-aminoisobutyric acid, l-BAIBA, is a muscle-derived osteocyte survival factor. Cell Rep.

[CR19] Krieger JM, Hagele AM, Orr LS et al. Dose-response absorption kinetics of oral L-Beta-aminoisobutyric acid (L-BAIBA) supplementation in healthy men and women. J Diet Suppl 2022:1–18. 10.1080/19390211.2022.2128141 36184601

[CR20] Kuratani T, Matsuda H, Sawa Y (1992). Experimental study in a rabbit model of ischemia-reperfusion lung injury during cardiopulmonary bypass. J Thorac Cardiovasc Surg.

[CR21] Li T, Zhang Z, Kolwicz SC (2017). Defective branched-chain amino acid catabolism disrupts glucose metabolism and sensitizes the heart to Ischemia-Reperfusion Injury. Cell Metab.

[CR23] Li Y, Xiong Z, Yan W (2020). Branched chain amino acids exacerbate myocardial ischemia/reperfusion vulnerability via enhancing GCN2/ATF6/PPAR-alpha pathway-dependent fatty acid oxidation. Theranostics.

[CR22] Li Y, Yang Y, Yang Y (2022). Multifaceted roles of ferroptosis in Lung Diseases. Front Mol Biosci.

[CR24] Lian K, Guo X, Wang Q (2020). PP2Cm overexpression alleviates MI/R injury mediated by a BCAA catabolism defect and oxidative stress in diabetic mice. Eur J Pharmacol.

[CR25] Luo W, Tao Y, Chen S (2022). Rosmarinic Acid ameliorates Pulmonary Ischemia/Reperfusion Injury by activating the PI3K/Akt signaling pathway. Front Pharmacol.

[CR27] Orrenius S, Gogvadze V, Zhivotovsky B (2007). Mitochondrial oxidative stress: implications for cell death. Annu Rev Pharmacol Toxicol.

[CR28] Pan Y, Wang X, Liu X et al. Targeting ferroptosis as a Promising Therapeutic Strategy for Ischemia-Reperfusion Injury. Antioxid (Basel) 2022; 11 (11). 10.3390/antiox11112196 PMC9686892 36358568

[CR29] Prieto P, Cuenca J, Traves PG (2010). Lipoxin A4 impairment of apoptotic signaling in macrophages: implication of the PI3K/Akt and the ERK/Nrf-2 defense pathways. Cell Death Differ.

[CR30] Roberts LD, Bostrom P, O’Sullivan JF (2014). beta-aminoisobutyric acid induces browning of white fat and hepatic beta-oxidation and is inversely correlated with cardiometabolic risk factors. Cell Metab.

[CR31] Sawada M, Yamamoto H, Ogasahara A (2019). beta-aminoisobutyric acid protects against vascular inflammation through PGC-1beta-induced antioxidative properties. Biochem Biophys Res Commun.

[CR32] Shanmugasundaram D, Fan Q, Wang M (2022). Safety Assessment of L-beta-aminoisobutyric acid (L-BAIBA): Subchronic Toxicity Study in Sprague Dawley rats. Int J Toxicol.

[CR34] Shi CX, Zhao MX, Shu XD (2016). beta-aminoisobutyric acid attenuates hepatic endoplasmic reticulum stress and glucose/lipid metabolic disturbance in mice with type 2 diabetes. Sci Rep.

[CR35] Smith KM, Mrozek JD, Simonton SC (1997). Prolonged partial liquid ventilation using conventional and high-frequency ventilatory techniques: gas exchange and lung pathology in an animal model of respiratory distress syndrome. Crit Care Med.

[CR36] Ucar BI, Ucar G, Saha S et al. Pharmacological protection against Ischemia-Reperfusion Injury by regulating the Nrf2-Keap1-ARE signaling pathway. Antioxid (Basel) 2021; 10 (6). 10.3390/antiox10060823 PMC8224095 34063933

[CR40] Wang Z, Chen Z, Jiang Z (2019). Cordycepin prevents radiation ulcer by inhibiting cell senescence via NRF2 and AMPK in rodents. Nat Commun.

[CR38] Wang Y, Quan F, Cao Q (2021). Quercetin alleviates acute kidney injury by inhibiting ferroptosis. J Adv Res.

[CR37] Wang X, Chen X, Zhou W (2022). Ferroptosis is essential for diabetic cardiomyopathy and is prevented by sulforaphane via AMPK/NRF2 pathways. Acta Pharm Sin B.

[CR39] Wang Y, Chen Z, Luo J (2023). Salidroside postconditioning attenuates ferroptosis-mediated lung ischemia-reperfusion injury by activating the Nrf2/SLC7A11 signaling axis. Int Immunopharmacol.

[CR41] Xiao Y, Xia J, Wu S et al. Curcumin Inhibits Acute Vascular Inflammation through the Activation of Heme Oxygenase-1. Oxid Med Cell Longev 2018; 2018:3295807. 10.1155/2018/3295807 PMC6171216 30327711

[CR42] Xingyue L, Shuang L, Qiang W (2021). Chrysin ameliorates Sepsis-Induced Cardiac Dysfunction through Upregulating Nfr2/Heme oxygenase 1 pathway. J Cardiovasc Pharmacol.

[CR43] Ye Y, Li X, Feng G (2022). 3,3’-Diindolylmethane induces ferroptosis by BAP1-IP3R axis in BGC-823 gastric cancer cells. Anticancer Drugs.

[CR44] Yu S, Jia J, Zheng J (2021). Recent progress of ferroptosis in Lung Diseases. Front Cell Dev Biol.

[CR45] Yu Y, Yan Y, Niu F (2021). Ferroptosis: a cell death connecting oxidative stress, inflammation and cardiovascular diseases. Cell Death Discov.

[CR46] Zeng C, Lin J, Zhang K et al. SHARPIN promotes cell proliferation of cholangiocarcinoma and inhibits ferroptosis via p53/SLC7A11/GPX4 signaling. Cancer Sci 2022. 10.1111/cas.15531 PMC9633309 35968603

[CR47] Zhang Y, Wu Q, Liu J (2022). Sulforaphane alleviates high fat diet-induced insulin resistance via AMPK/Nrf2/GPx4 axis. Biomed Pharmacother.

