# Supplementary figures and images for: β-aminoisobutyrics acid, a metabolite of BCAA, activates the AMPK/Nrf-2 pathway to prevent ferroptosis and ameliorates lung ischemia-reperfusion injury
Source: Mol Med. 2023 Dec 4;29:164. doi: 10.1186/s10020-023-00729-z (PMC10696792; doi:10.1186/s10020-023-00729-z)

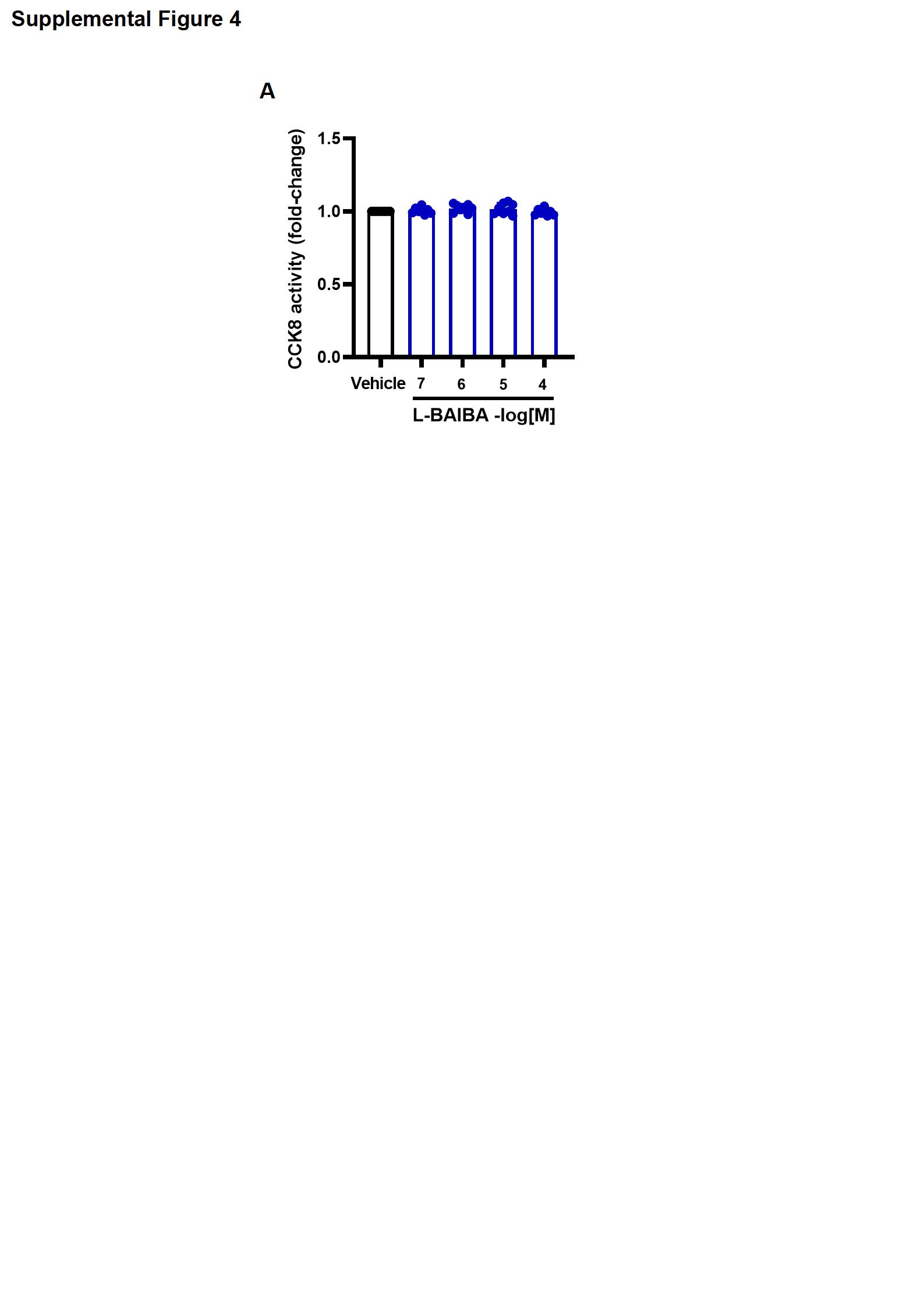

Supplement: Supplementary file 1 — Supplementary Material 1 [file 10020_2023_729_MOESM1_ESM.jpg]

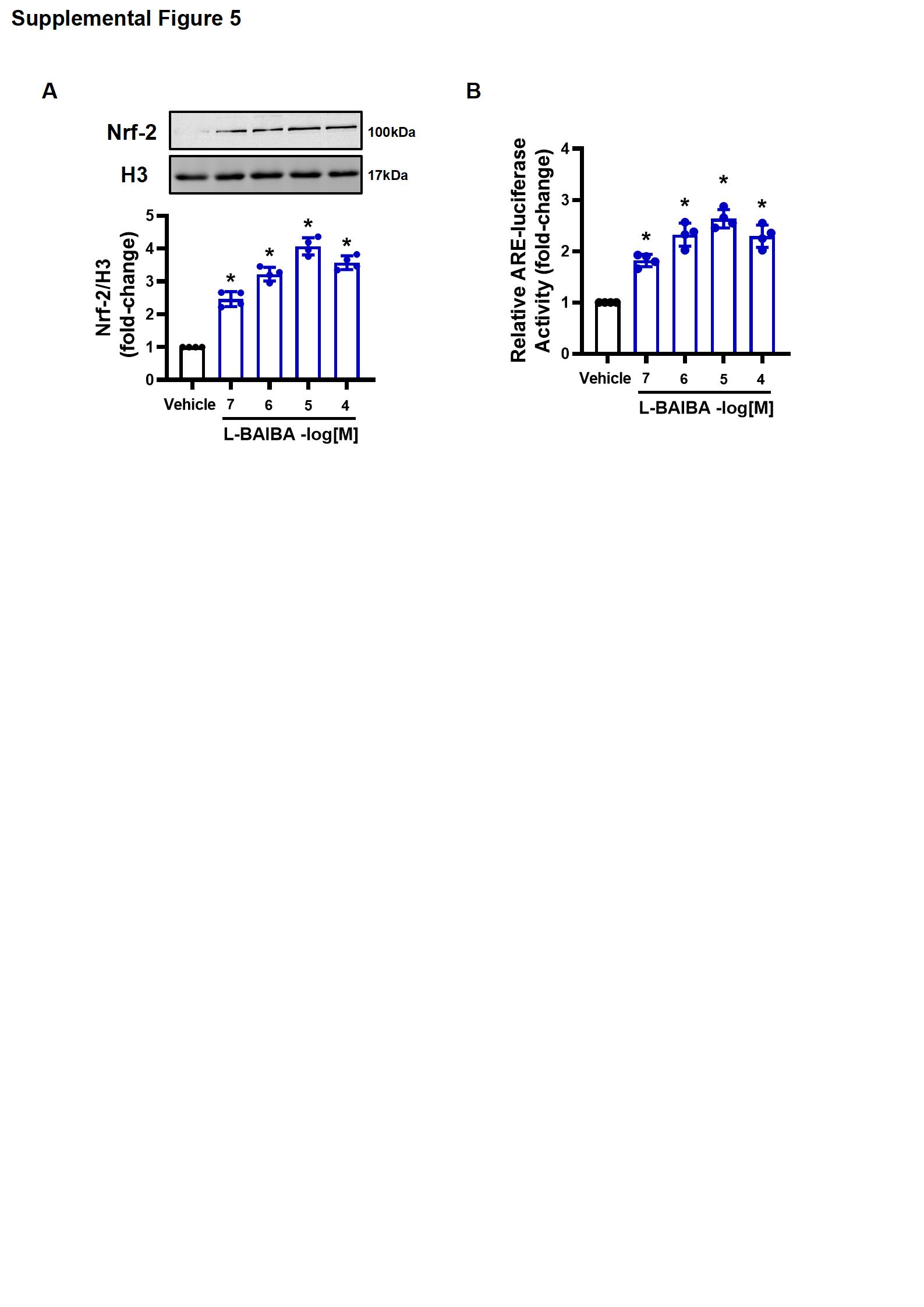

Supplement: Supplementary file 2 — Supplementary Material 2 [file 10020_2023_729_MOESM2_ESM.jpg]

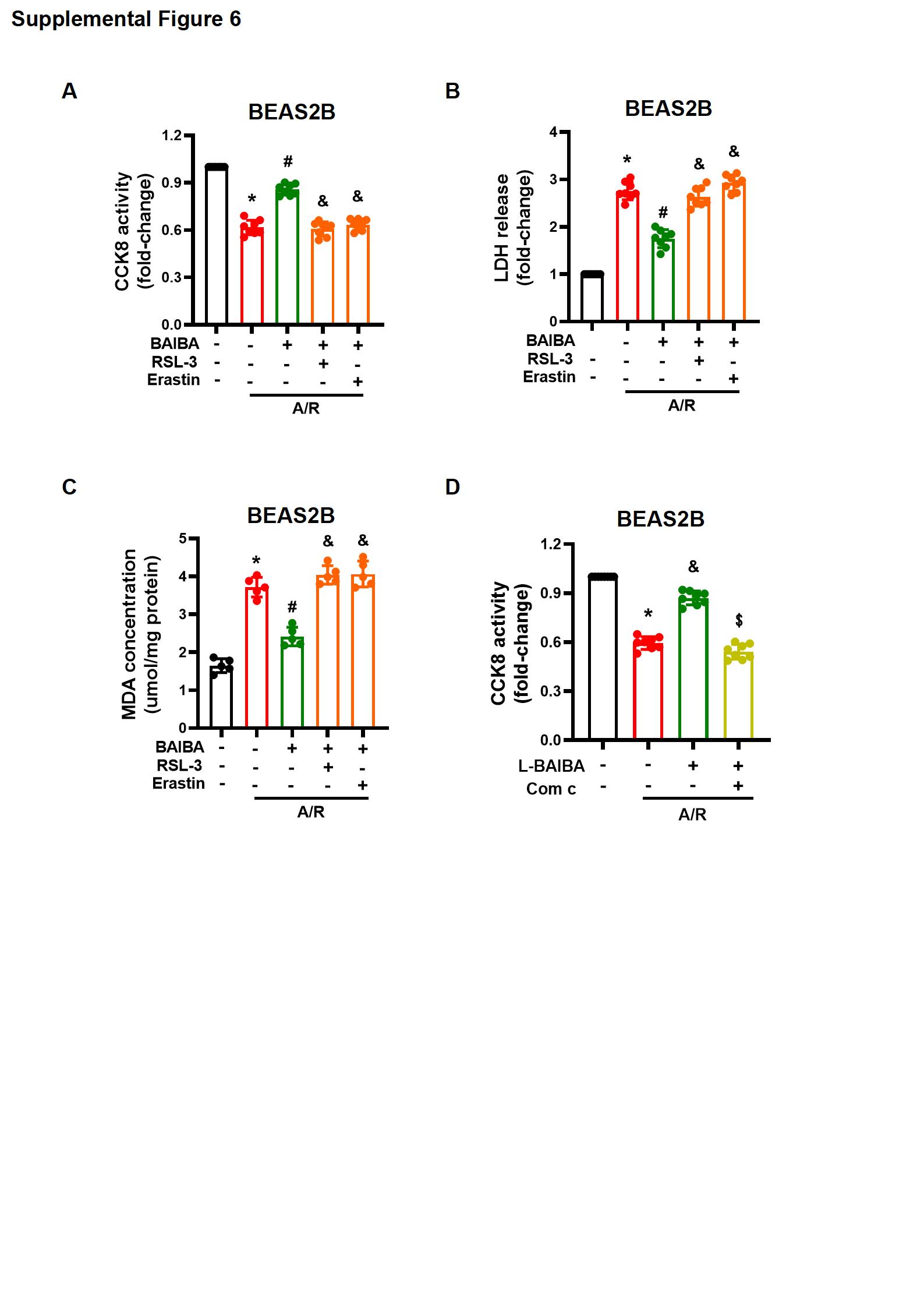

Supplement: Supplementary file 3 — Supplementary Material 3 [file 10020_2023_729_MOESM3_ESM.jpg]

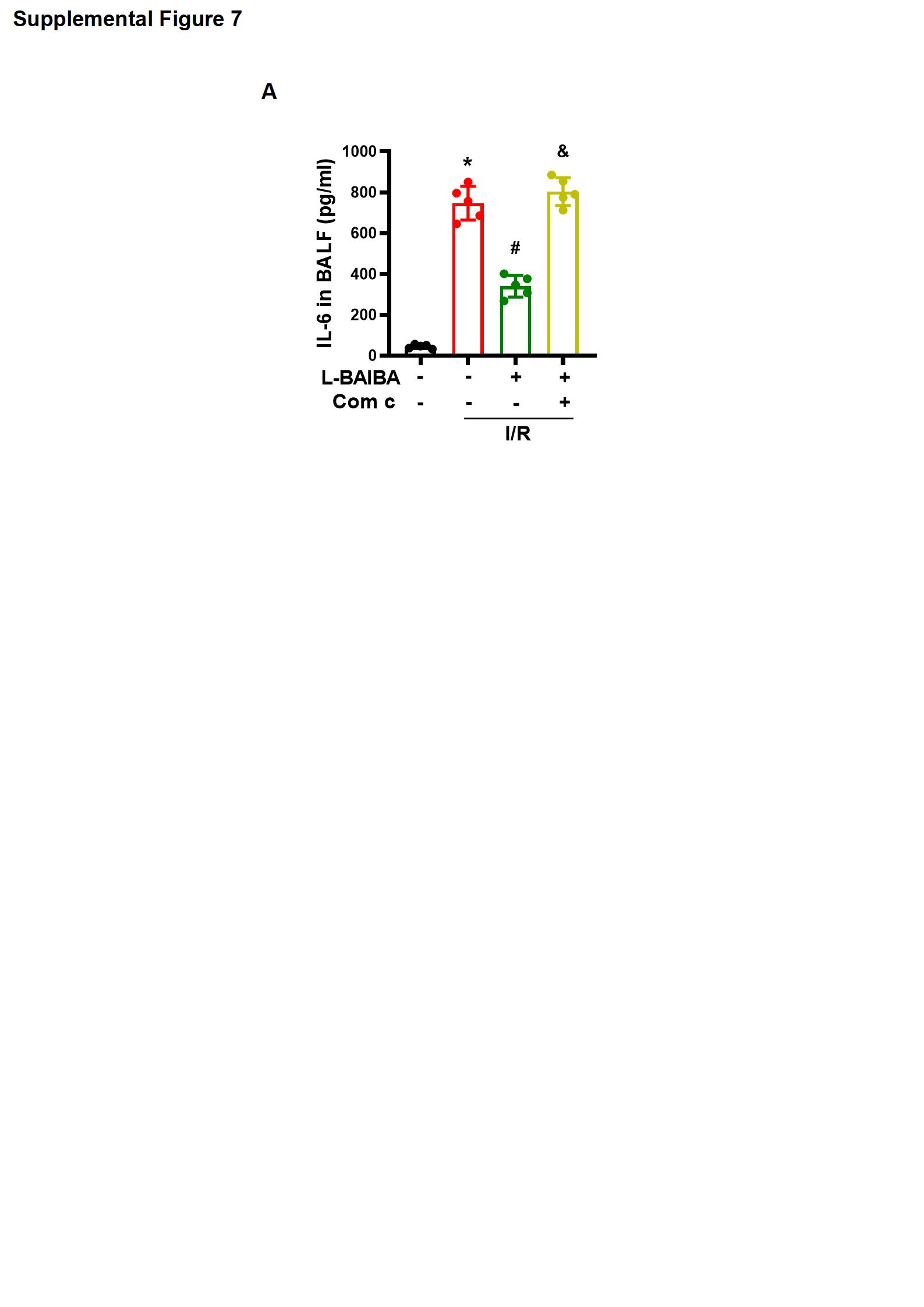

Supplement: Supplementary file 4 — Supplementary Material 4 [file 10020_2023_729_MOESM4_ESM.jpg]

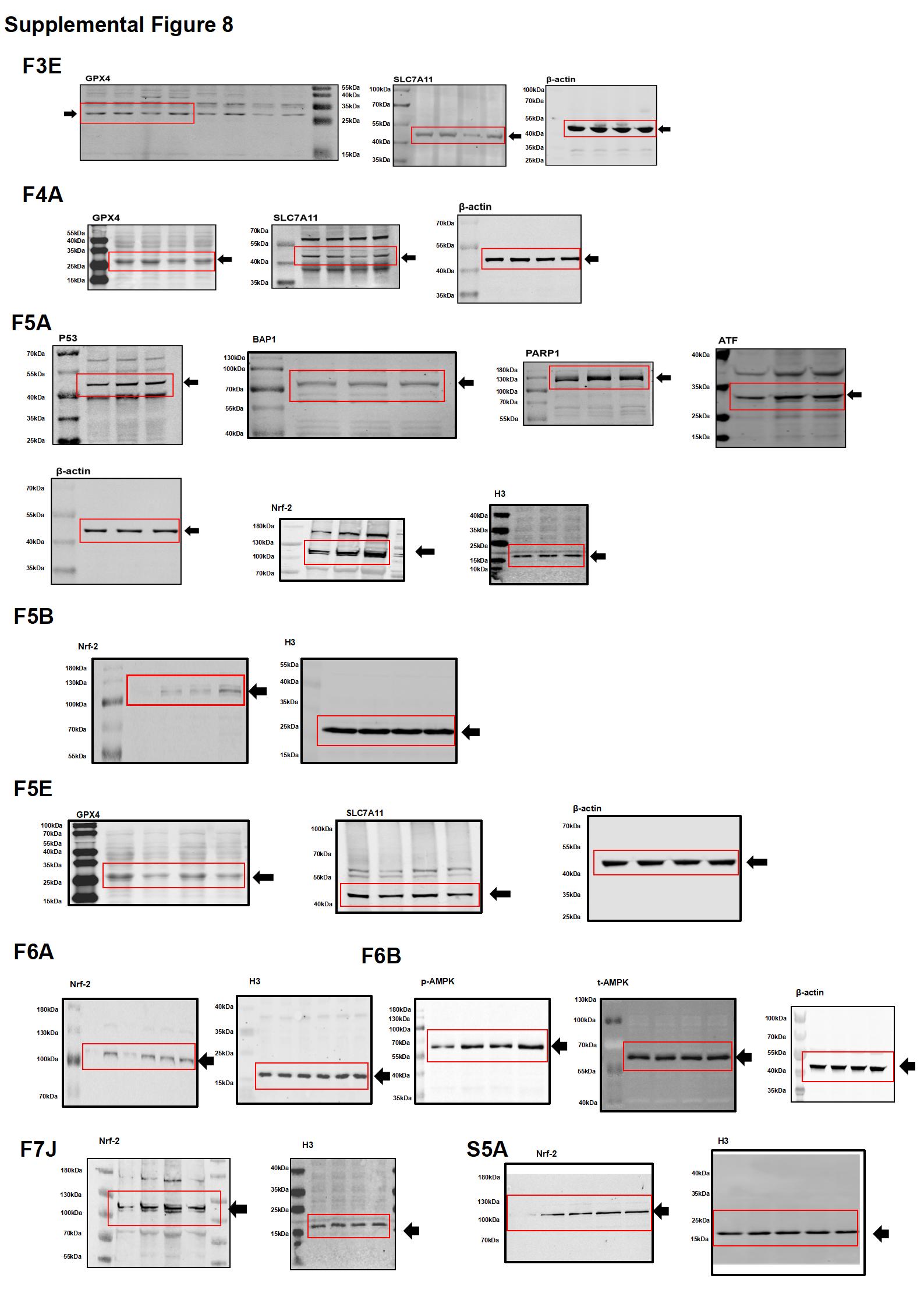

Supplement: Supplementary file 5 — Supplementary Material 5 [file 10020_2023_729_MOESM5_ESM.jpg]

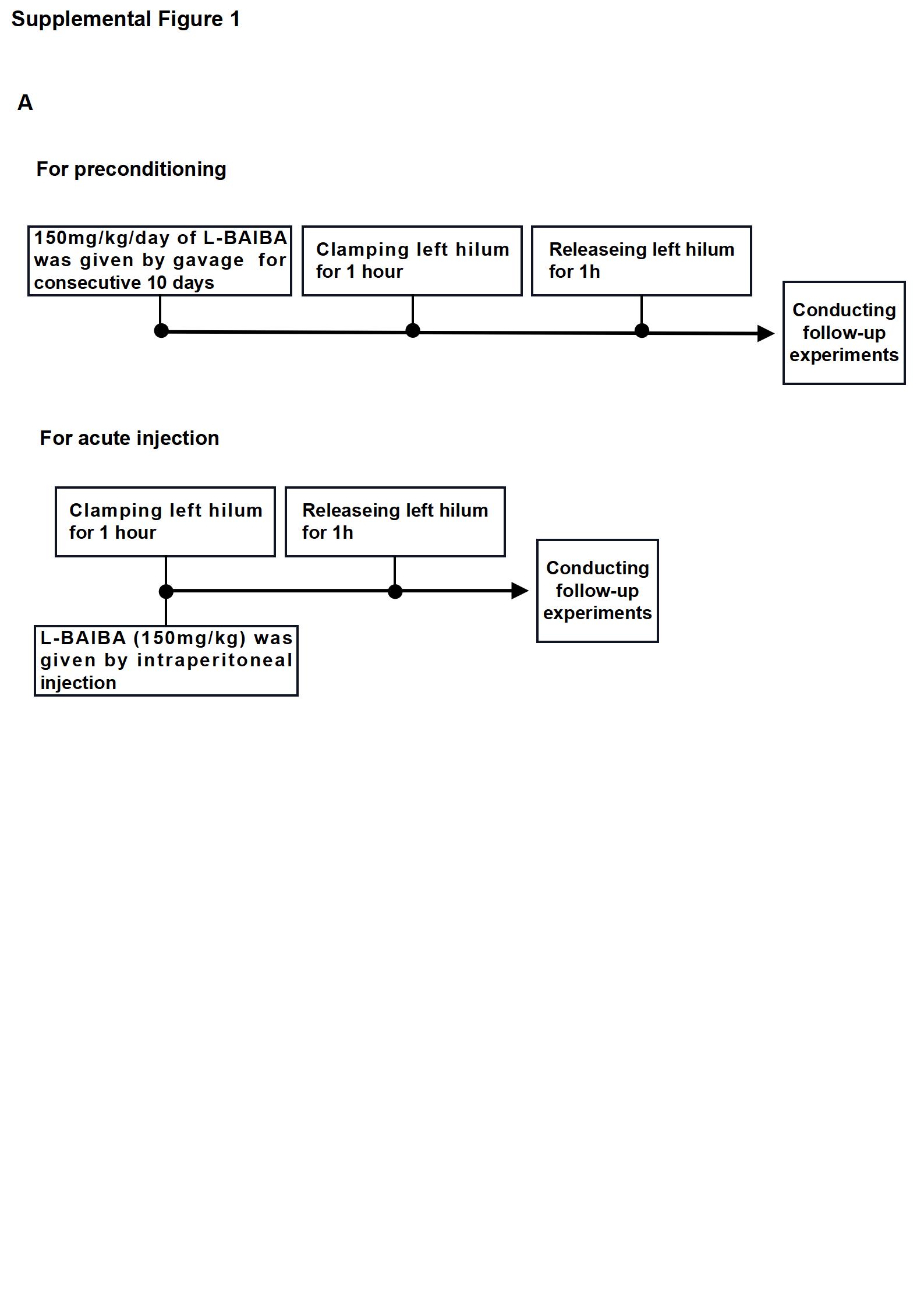

Supplement: Supplementary file 6 — Supplementary Material 6 [file 10020_2023_729_MOESM6_ESM.jpg]

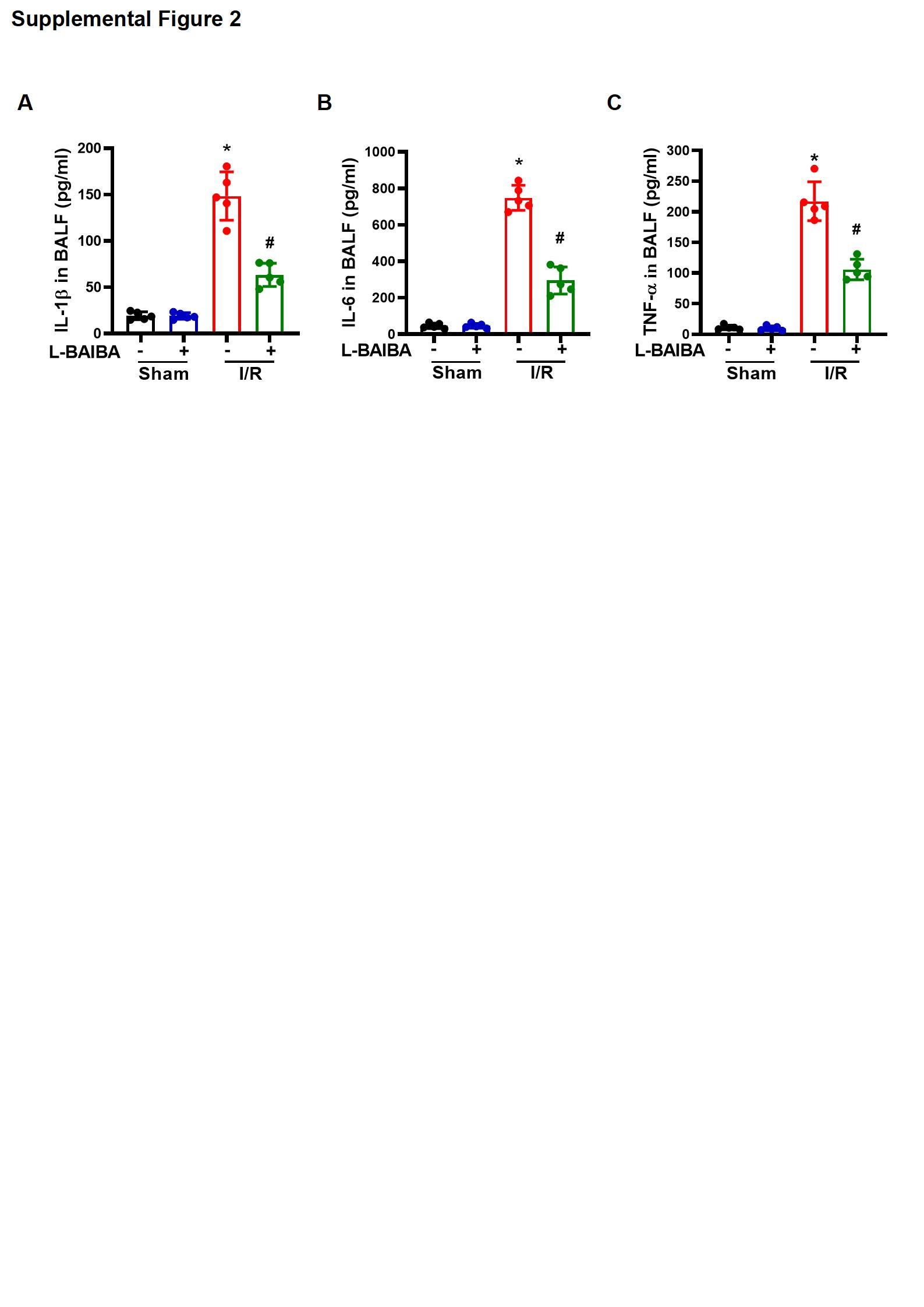

Supplement: Supplementary file 7 — Supplementary Material 7 [file 10020_2023_729_MOESM7_ESM.jpg]

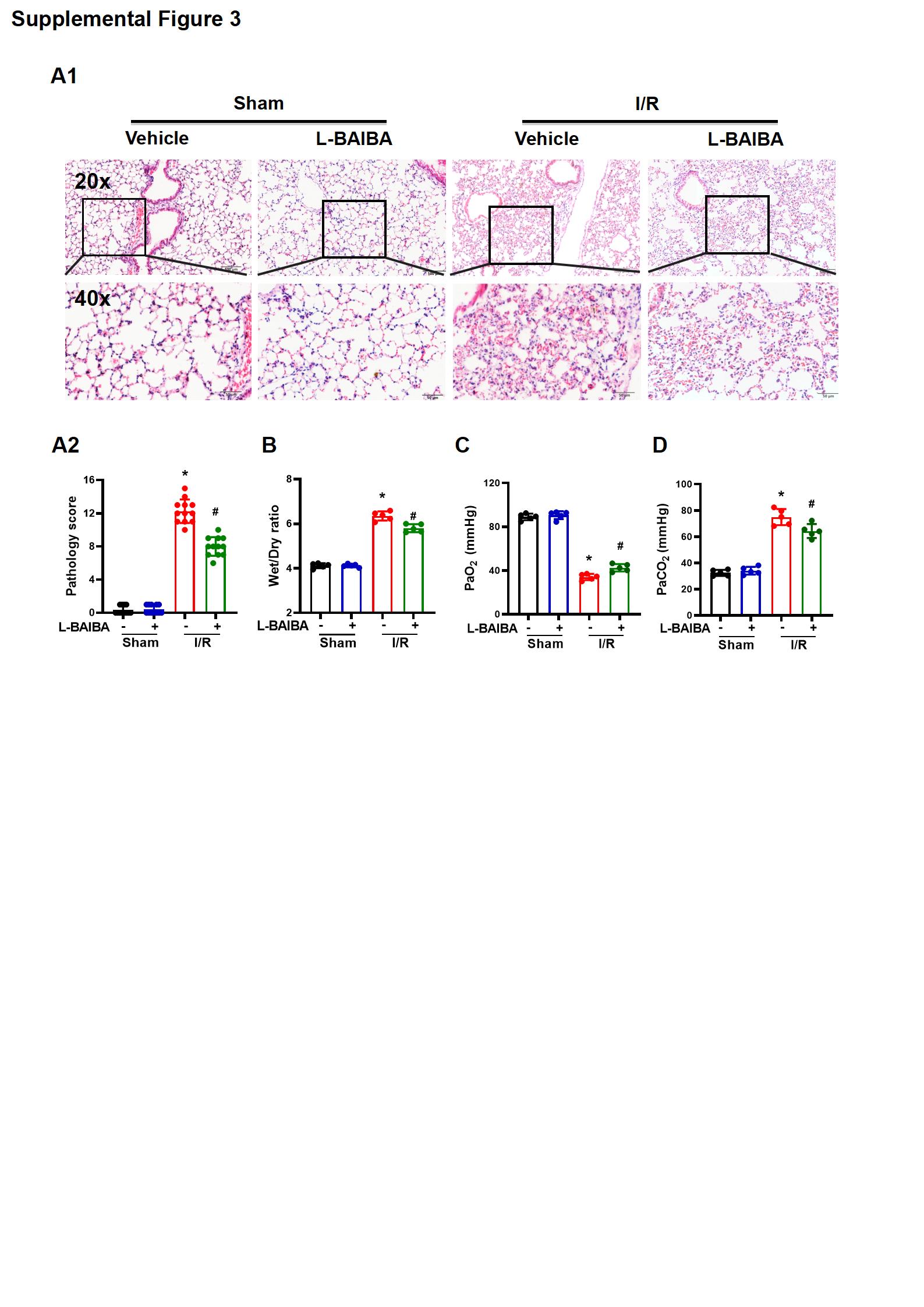

Supplement: Supplementary file 8 — Supplementary Material 8 [file 10020_2023_729_MOESM8_ESM.jpg]
