# Supplementary material for: β-aminoisobutyrics acid, a metabolite of BCAA, activates the AMPK/Nrf-2 pathway to prevent ferroptosis and ameliorates lung ischemia-reperfusion injury
Source: Mol Med. 2023 Dec 4;29:164. doi: 10.1186/s10020-023-00729-z (PMC10696792; doi:10.1186/s10020-023-00729-z)
Supplement: Supplementary file 9 — Supplementary Material 9 [file 10020_2023_729_MOESM9_ESM.docx]

**SUPPLEMENTAL IMFORMATION**

# β-aminoisobutyric Acid, a Metabolite of BCAA, Activates the AMPK/Nrf-2 Pathway to Prevent Ferroptosis and Ameliorates Lung Ischemia-Reperfusion Injury

Ziyue Zhang^1,2,3†^, Xingbing Li^1,2,4†^, Jingwen Guo^1,2^, Bo He^1,2^, Lianpan Wu^1,2^, Rongpei Yang^1,2^, Xingyue Li^2^, Dandong Fang^1,2^, XiaoLi Yang^1,2^, Donghai Yang^1,2^, Fengxian Wang^1,2^, Ming Tang^1,2^, Yu Han^1,2^, Pedro A. Jose^5^, Hongyong Wang*^1,2^, Chunyu Zeng*^1,2,6,7^

^1^Department of Cardiology, Daping Hospital, The Third Military Medical University (Army Medical University), Chongqing, P. R. China.

^2^Key Laboratory of Geriatric Cardiovascular and Cerebrovascular Disease Research, Ministry of Education of China; Chongqing Key Laboratory for Hypertension Research, Chongqing Cardiovascular Clinical Research Center, Chongqing Institute of Cardiology, Chongqing, P. R. China.

^3^Outpatient department, 96608 hospital of PLA, Hanzhong, Shanxi, P. R. China.

^4^Department of Cardiology, Chongqing Hospital of Traditional Chinese Medicine, Chongqing, P. R. China.

^5^Division of Renal Diseases& Hypertension, Department of Medicine and Department of Physiology/Pharmacology, The George Washington University School of Medicine & Health Sciences, Washington, DC

^6^State Key Laboratory of Trauma, Burns and Combined Injury, Daping Hospital, The Third Military Medical University (Army Medical University), Chongqing, P. R. China.

^7^Cardiovascular Research Center of Chongqing College, Chinese Academy of Sciences, University of Chinese Academy of Sciences, Chongqing, P. R. China.

**Corresponding Author:**

Correspondence should be addressed to Hongyong Wang, E-mail: [whysir@aliyun.com](mailto:whysir@aliyun.com) and Chunyu Zeng, E-mail: zengchunyu@tmmu.edu.cn.

† These authors made equal contributions to this work.


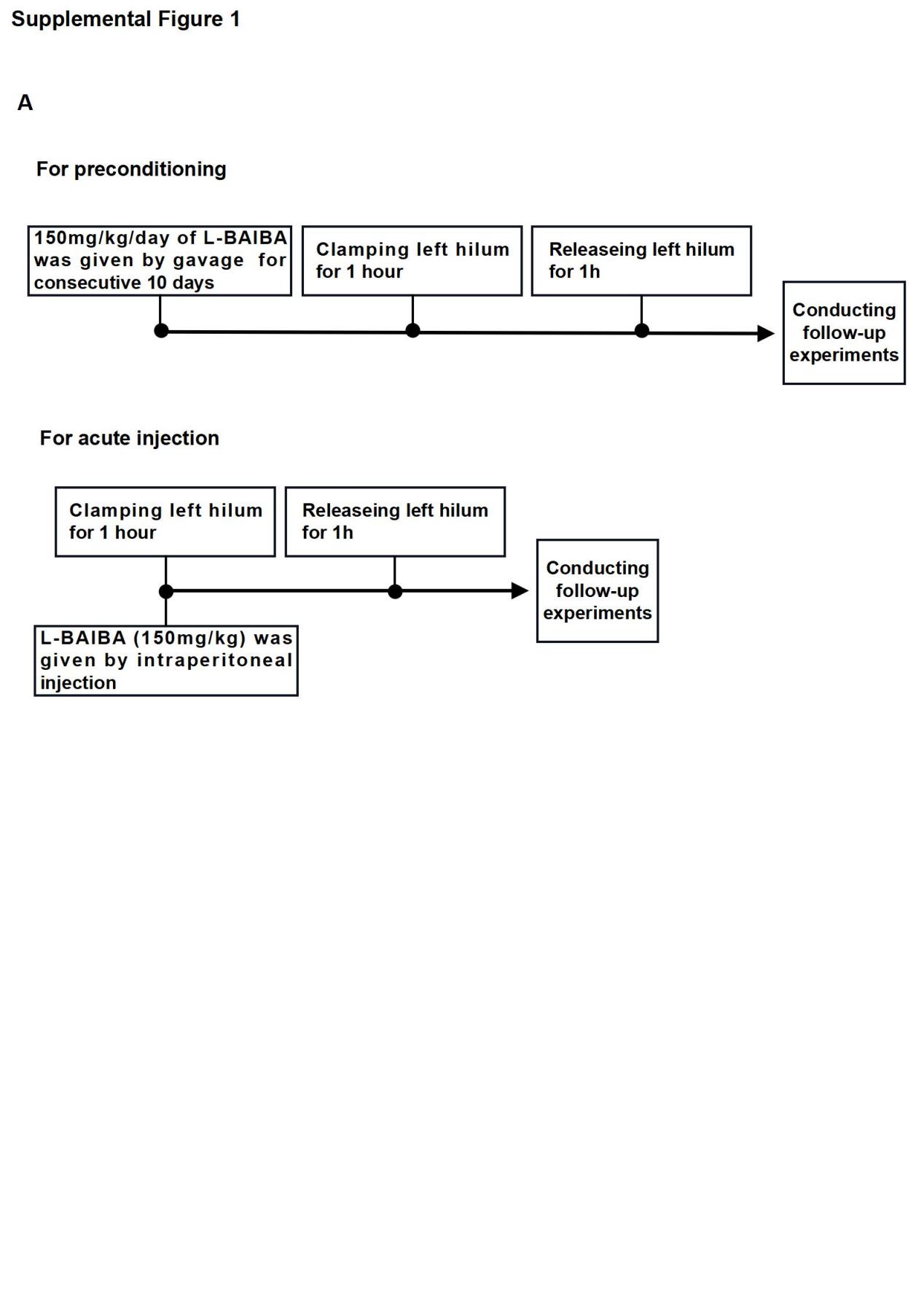


**Supplemental Figure 1. Treatment scheme of L-BAIBA.**

(**A**): For preconditioning, mice was given L-BAIBA (150mg/kg/day) in the drinking water by gavage for 10 consecutive days. Then, lung I/R injury models were established. For acute injection, L-BAIBA (150mg/kg) was given by intraperitoneal injection immediately after lung ischemia.

**
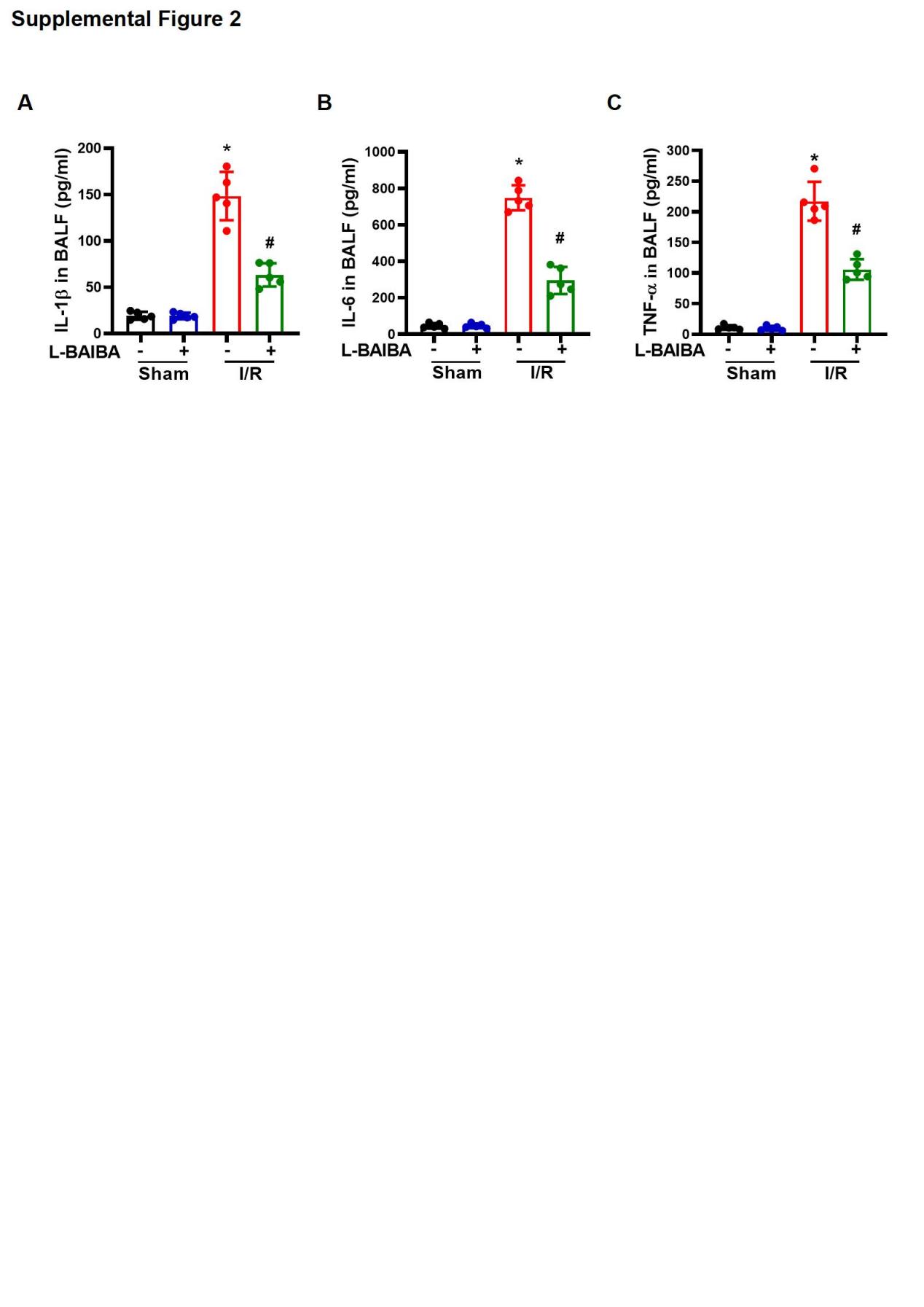
**

**Supplemental Figure 2. L-BAIBA inhibits inflammation in lung I/R injury.**

(**A-C**): Concentrations of interleukin-1β (IL-1β) (**A**), IL-6 (**B**) and TNF-α (**C**) in BALF were measured by ELISA (n=5). The values are presented as mean±SD (*P<0.05 vs. Sham, #P<0.05 vs. I/R only, Kolmogorov-Smirnov test was used for normality, ANOVA was used for statistical analysis and post hoc analysis was performed by using Tamhane test (**A**, **B**) or LSD (**C**)).


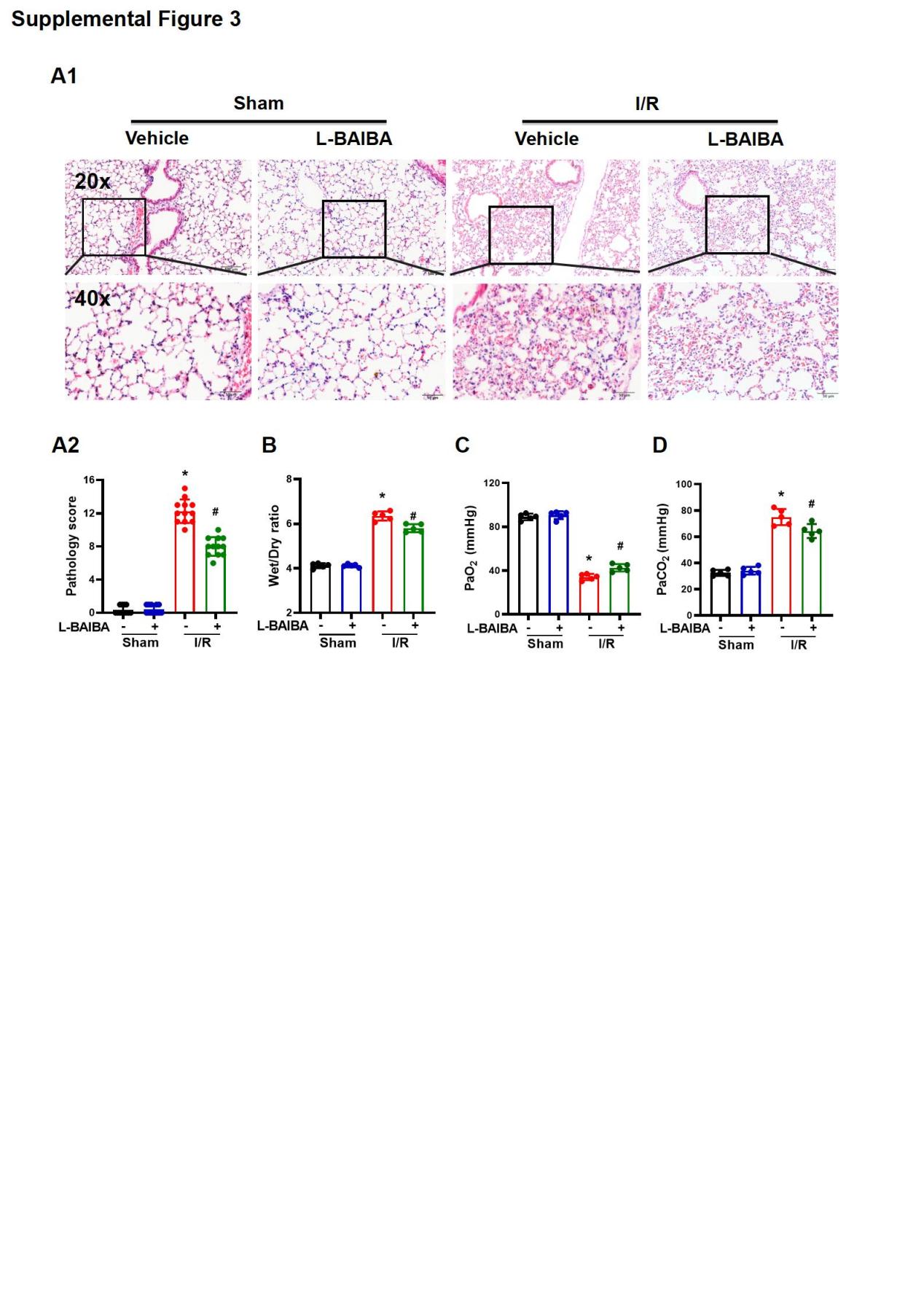


**Supplemental Figure 3. Acute administration of L-BAIBA can effectively protect against lung I/R injury.**

(**A**): Histopathological images of I/R-induced lung injury in mouse with or without L-BAIBA administration, representative images was shown and statistical results of the pathological score were showed (n=12). (**B**): The degree of lung edema in mouse was evaluated by the ratio of lung wet and dry weight (n=5). (**C** and **D**): The arterial blood PaO2 (**C**) and PaCO2 (**D**) were measured to evaluated the pulmonary oxygenation (n=5). The values are presented as mean±SD (*P < 0.05 vs. Sham, #P<0.05 vs. I/R only, Kolmogorov-Smirnov test was used for normality, ANOVA was used for statistical analysis and post hoc analysis was performed by using Tamhane test (**A**) or LSD (**B**, **C**, **D**)).


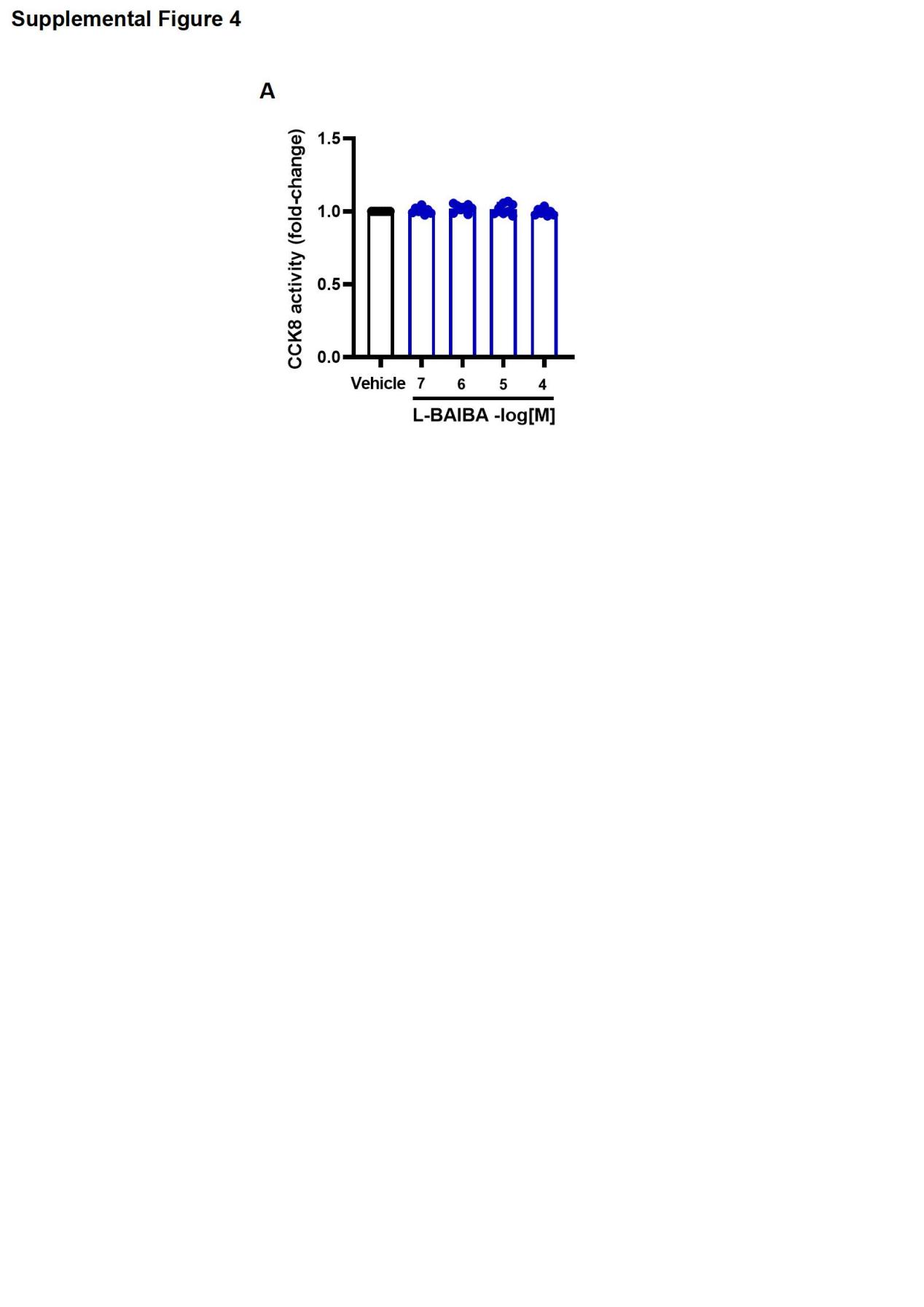


**Supplemental Figure 4. L-BAIBA in the physiological state is not toxic to A549 cells.**

**(A)**: Effect of L-BAIBA on A549 cell activities under normoxic condition, indicated by CCK-8 assay (n=8). The values are presented as mean ± standard deviation. Kolmogorov-Smirnov test was used for normality, ANOVA was used for statistical analysis and post hoc analysis was performed by using Tamhane test.

**
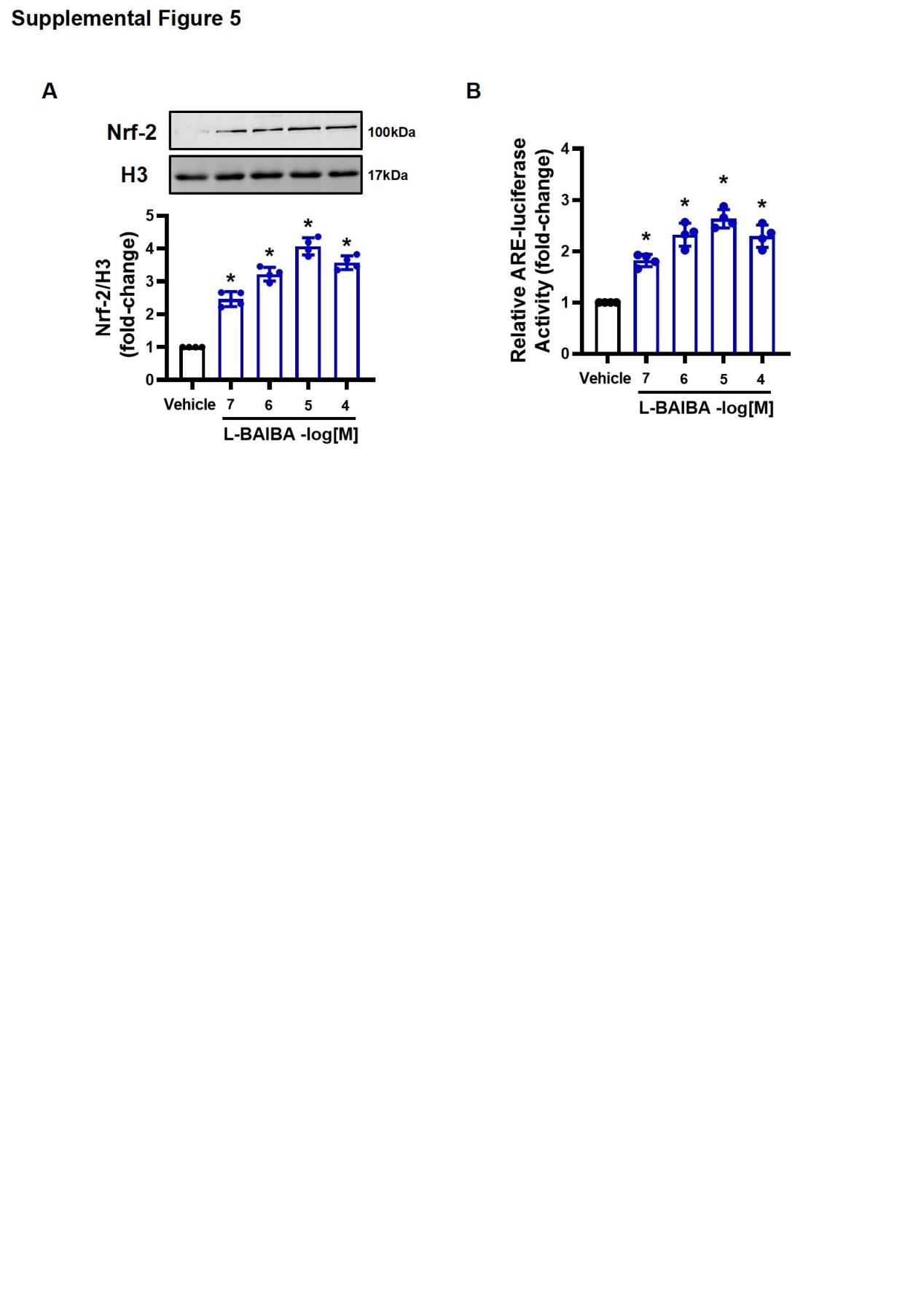
Supplemental Figure 5. L-BAIBA activates Nrf-2 signaling pathway and enhance cells antioxidant abilities.**

**(A)**: L-BAIBAincreased nuclear Nrf-2 levels in a concentration-dependent manner in A549 cells (n=4).**(B)**: The antioxidant responseelement (ARE) luciferase reporter gene was used to confirm the activate of Nrf-2 signaling pathway (n=4).The values are presented as mean ± standard deviation (*P<0.05 vs.Control, Kolmogorov-Smirnov test was used for normality, ANOVA was used for statistical analysis and post hoc analysis was performed by using Tamhane test (**A**) or LSD (**B**)).


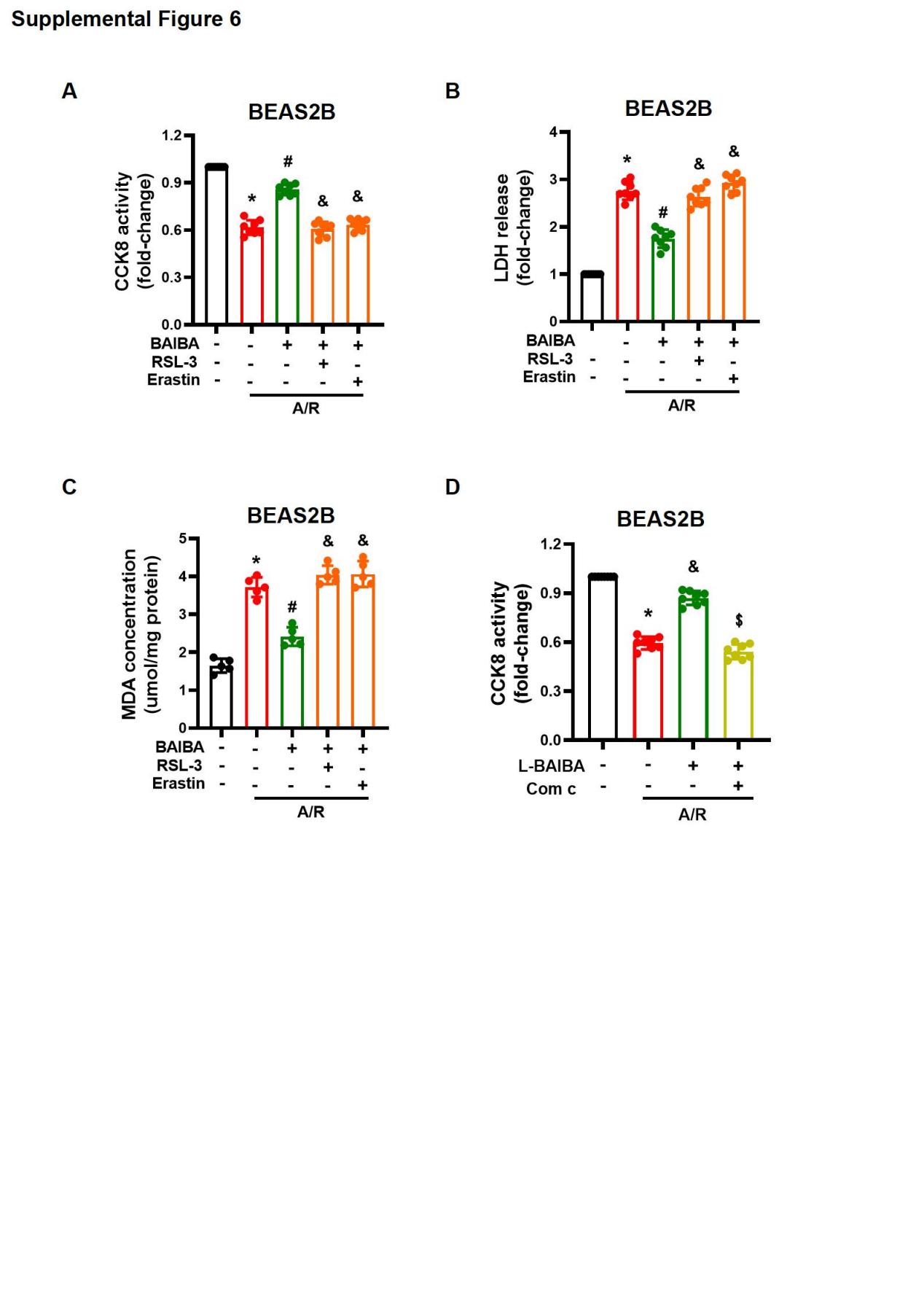


**Supplemental Figure 6. L-BAIBA can protect against A/R-induced injury in BEAS-2B cells by inhibiting ferroptosis.**

**(A** and **B)**: Effects of L-BAIBA on cell activity exposed to A/R injury were determined by CCK8 experiments and the release of LDH in BEAS-2B cells (n=8).**(C)**: The concentrations of MDA were measured (n=5). **(D)**: Results of CCK8 assays in BEAS-2B cells, with or without compound c treatment (n=8). The values are presented as mean ± SD (*P<0.05 vs. Control, #P<0.05 vs. A/R only, &P<0.05 vs. L-BAIBA+A/R, Kolmogorov-Smirnov test was used for normality,ANOVA was used for statistical analysis and post hoc analysis was performed by using Tamhane test (**A**, **B**, **D**) or LSD (**C**)).


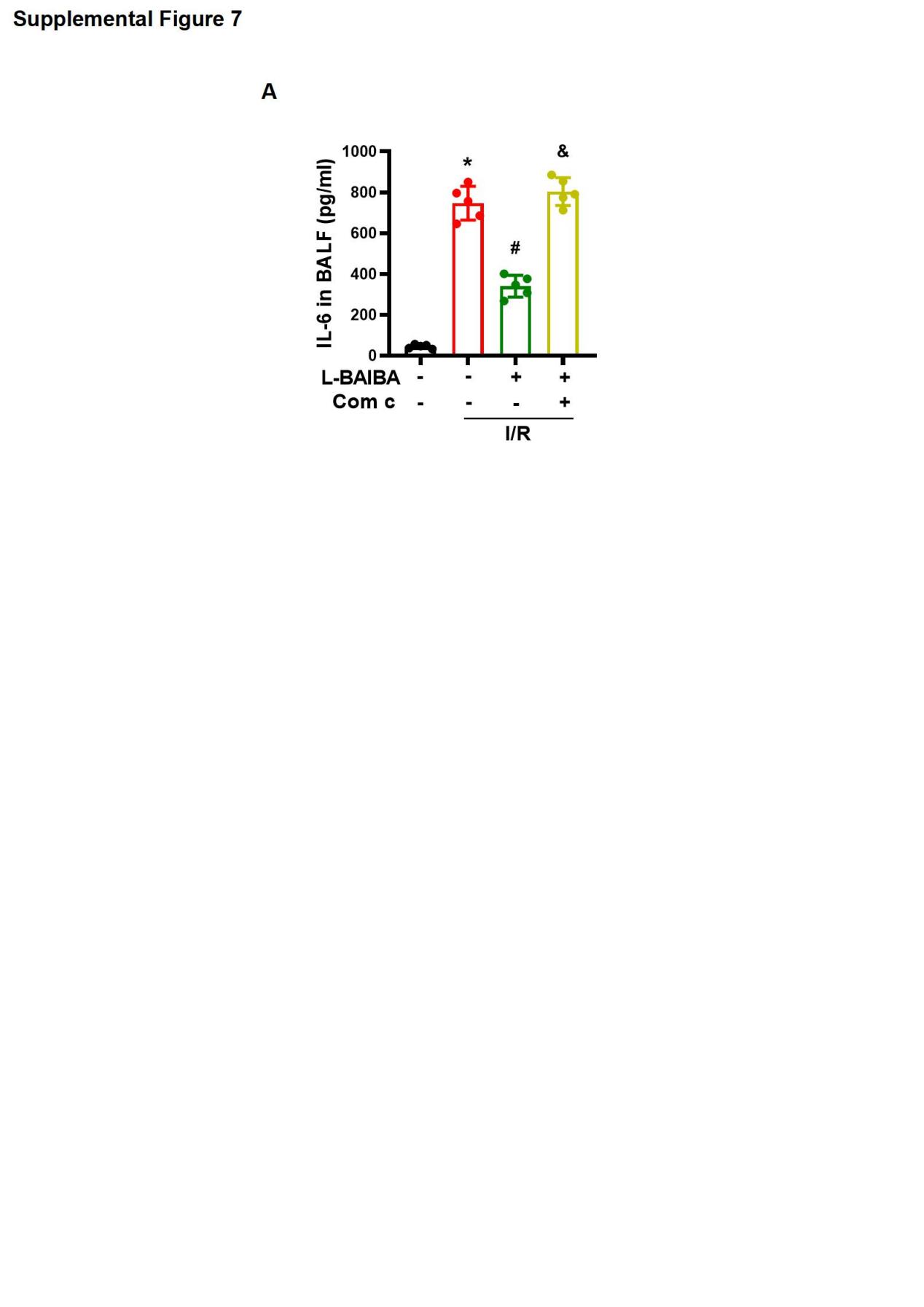


**Supplemental Figure 7. Compound c eliminates the inhibitory effects of L-BAIBA on inflammation induced by I/R.**

**(A)**: Concentrations of IL-6 in BALF were measured by ELISA (n=5).The values are presented as mean± standard deviation (*P < 0.05 vs.Sham, #P<0.05 vs.I/R only, &P<0.05 vs.L-BAIBA+I/R, Kolmogorov-Smirnov test was used for normality, ANOVA was used for statistical analysis and post hoc analysis was performed by using LSD).


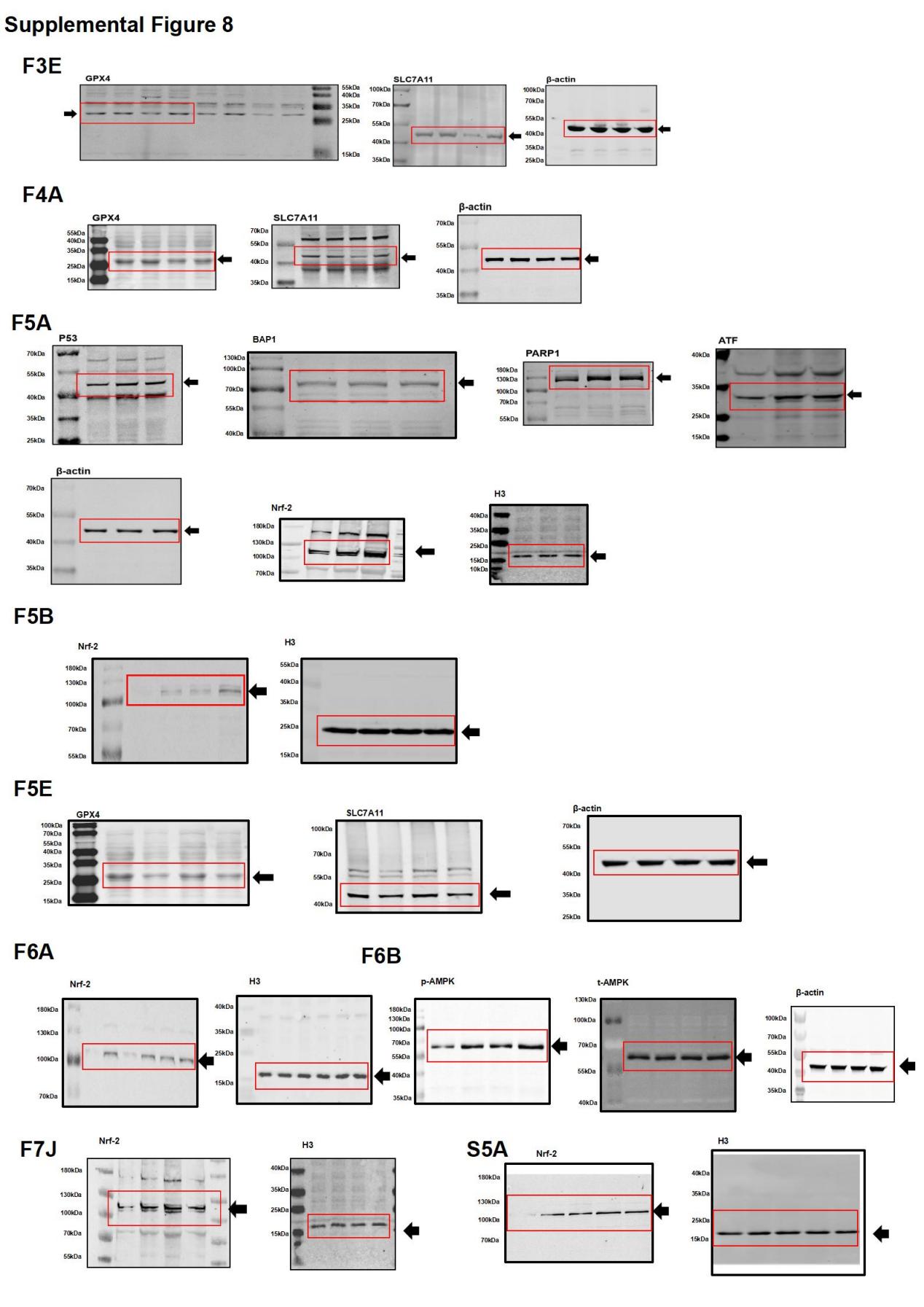


**Supplemental Figure 8. Raw images of western blot.**
