# Supplementary material for: β-aminoisobutyrics acid, a metabolite of BCAA, activates the AMPK/Nrf-2 pathway to prevent ferroptosis and ameliorates lung ischemia-reperfusion injury
Source: Mol Med. 2023 Dec 4;29:164. doi: 10.1186/s10020-023-00729-z (PMC10696792; doi:10.1186/s10020-023-00729-z)
Supplement: Supplementary file 10 — Supplementary Material 10 [file 10020_2023_729_MOESM10_ESM.docx]

July 14, 2023

Dear Dr. Rosamaria Ruggieri:

We thank you for giving us the opportunity to respond to the reviewers’ insightful comments and suggestions. On behalf of all the co-authors, I am re-submitting our revised manuscript “β-aminoisobutyric Acid, a Metabolite of BCAA, Activates the AMPK/Nrf-2 Pathway to Prevent Ferroptosis and Ameliorates Lung Ischemia-Reperfusion Injury” (Manuscript ID: MOME-D-23-00149) for consideration for publication in Molecular Medicine.

As per the recommendations of the reviewers, we added the key data on the effect of L-BAIBA on anoxia/reoxygenation (A/R) in non-malignant lung cells (BEAS-2B) and the *in vivo* study on the acute treatment of ischemia/reperfusion (I/R)-induced lung injury. Our point-by-point responses to the reviewers’ critiques are in a separate document.

We wish again to thank the reviewers for their guidance. We also thank you for handling our submitted manuscript. We look forward to hearing back from you.

Yours sincerely,

Chunyu Zeng, M.D., Ph.D.

Professor in Cardiology

Chair, Department of Cardiology

Daping Hospital, The Third Military Medical University

E-mail: zengchunyu@tmmu.edu.cn
